# RNA-Polymer Hybrids via Direct and Site-Selective Acylation with the ATRP Initiator and Photoinduced Polymerization

**DOI:** 10.1021/jacs.3c03757

**Published:** 2023-06-26

**Authors:** Jaepil Jeong, Xiaolei Hu, Hironobu Murata, Grzegorz Szczepaniak, Marta Rachwalak, Anna Kietrys, Subha R. Das, Krzysztof Matyjaszewski

**Affiliations:** † Department of Chemistry, Carnegie Mellon University, Pittsburgh, Pennsylvania 15213, United States; ‡ Center for Nucleic Acids Science & Technology, Carnegie Mellon University, Pittsburgh, Pennsylvania 15213, United States; § Faculty of Chemistry, University of Warsaw, Pasteura 1, 02-093 Warsaw, Poland

## Abstract

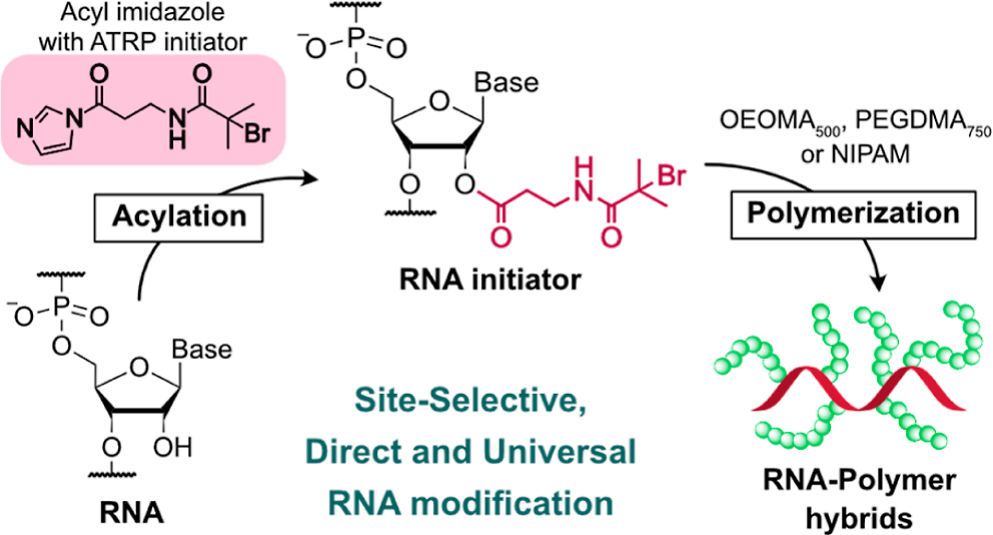

Combining synthetic polymers with RNA paves the way for creating RNA-based materials with non-canonical functions. We have developed an acylation reagent that allows for direct incorporation of the atom transfer radical polymerization (ATRP) initiator into both short synthetic oligoribonucleotides and natural biomass RNA extracted from torula yeast. The acylation was performed in a quantitative yield. The resulting initiator-functionalized RNAs were used for grafting polymer chains from the RNA by photoinduced ATRP, resulting in RNA-polymer hybrids with narrow molecular weight distributions. The RNA initiator was used for the polymerization of oligo(ethylene oxide) methyl ether methacrylate, poly(ethylene glycol) dimethacrylate, and *N*-isopropylacrylamide monomers, resulting in RNA bottlebrushes, hydrogels, and stimuli-responsive materials. This approach, readily applicable to both post-synthetic and nature-derived RNA, can be used to engineer the properties of a variety of RNA-based macromolecular hybrids and assemblies providing access to a wide variety of RNA-polymer hybrids.

## Introduction

Nucleic acid-polymer hybrids have emerged as a new class of biomaterials.^1^ They combine programmable self-assembly and biocompatibility with the versatility and diversity of synthetic polymers bringing enhanced properties of the molecular chimeras.^2^ Recent insights gained from the engineering of DNA-based materials and DNA-polymer conjugates have been instrumental in guiding the development of RNA-based nanomaterials and RNA-polymer hybrids.^3,4^

The fabrication of the RNA-synthetic polymer hybrid materials has been facilitated through one of the following approaches: (1) noncovalent attachment of pre-synthesized polymers with RNA by electrostatic interactions^5^ or hydrogen bonding;^6,7^ (2) covalent grafting of pre-synthesized polymers onto RNA equipped with reactive handles through coupling reactions such as (strain-promoted or copper-catalyzed) azide–alkyne cycloadditions,^8−10^ amidation,^11^ or disulfide exchange;^12,13^ (3) covalent conjugation of a polymerization initiator or acrylate moiety into RNA structures and subsequent polymerization from the RNA macroinitiators;^14,15^ or grafting through RNA-acrylate macromonomers.^16,17^ Importantly, the noncovalent and the covalent coupling strategies have distinct advantages and disadvantages. For example, the noncovalent approaches may be applied to a broad range of RNA substrates regardless of the length, source, or structure of the RNA substrate for coupling. However, the weak and non-covalent interaction between nucleic acids and polymers often raises concerns about the stability of the complex as well as difficulties in control over the number- and binding site of polymers.^18−21^ In contrast, due to the specificity of the coupling chemistries, covalent modification methods allow for chemically stable and precise incorporation of pre-synthesized polymers, acrylate groups, or polymerization-initiating moieties into the predetermined sites in nucleic acids.^22,23^ However, coupling handles (e.g., alkyne, thiol, norbornene, amine, and so forth) must be incorporated into the nucleic acid through the solid-phase synthesis or the use of an enzyme, which limits the scope of the substrate and, thus, hinders practical and versatile application.^24,25^ Therefore, for the successful modification of RNA with polymers, we desired an approach that would be capable of both (i) direct and (ii) covalent modification of RNA, to circumvent the problems related to current synthetic methods. We also sought a universal approach that would expand the scope of RNA substrates from short synthetic oligonucleotides to longer RNA transcripts or biomass RNA (bmRNA) extracted from natural sources.^26,27^

**Figure 1:**
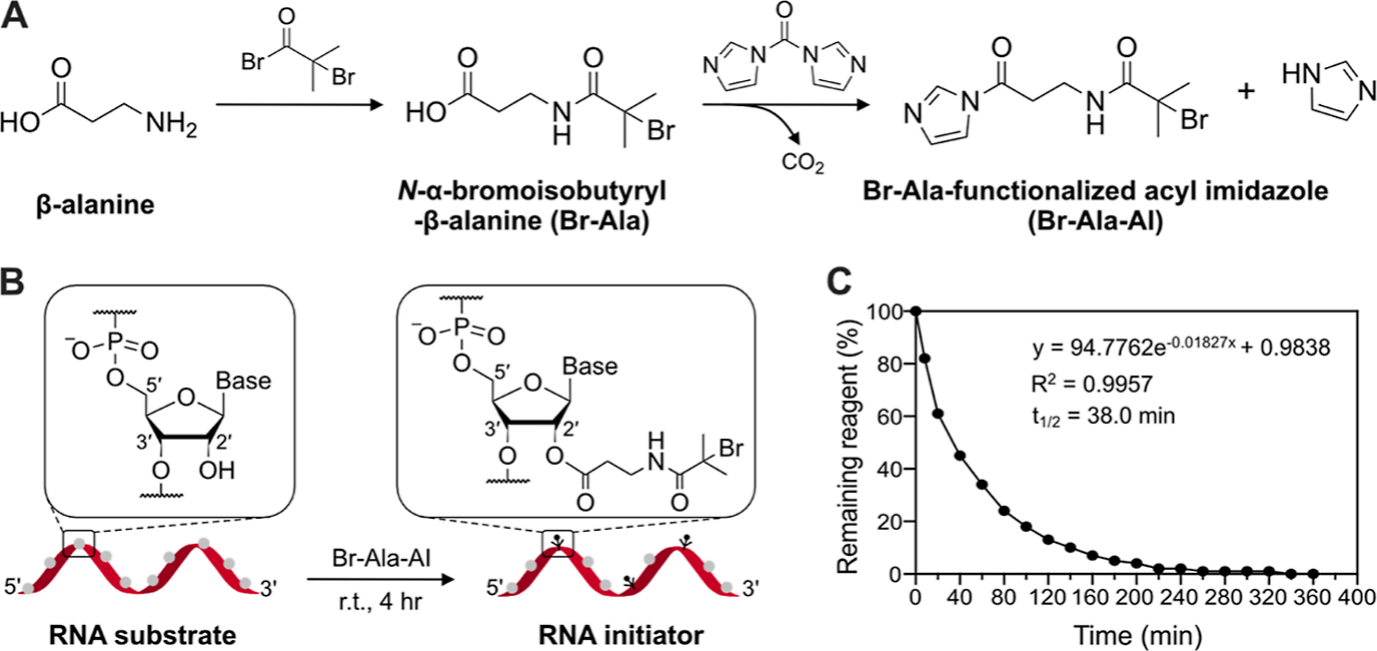
Synthesis of ATRP initiator-functionalized acyl imidazole reagent for RNA modification. (A) Scheme for the synthesis of **Br-Ala-AI**. (B) Schematic illustration of RNA functionalization with the **Br-Ala-AI** for the fabrication of RNA initiator. (C) Plot of hydrolysis kinetics of **Br-Ala-AI** reagent under the acylation condition (20% *v*/*v* DMSO in water). ^1^H NMR spectroscopy was used to monitor the hydrolysis of **Br-Ala-AI** and calculate the amount of remaining reagent (see Figure S2).

To address this challenge and to facilitate covalent RNA modification in a universal and direct manner, we explored the acyl imidazole chemistry: the activation of a carboxylic acid with an imidazole leaving group, which could covalently react with 2′-hydroxyl (2′-OH) groups in RNA to form 2′-O-adducts (see Figure 1). Indeed, acyl imidazole reagents have long been studied as structural mapping agents of RNA via selective 2′-hydroxyl acylation analyzed by primer extension (SHAPE).^28,29^ In addition, compared to other reagents that are known to react with nucleic acids (e.g., dimethyl sulfate, ninhydrin, psoralen, diazo compounds, and so forth), acyl imidazole reagents have significant advantages, such as high reaction yield and site-selective incorporation, while generating a less toxic and biocompatible by-product (i.e., imidazole) that allows RNA mapping even in vitro^30^ and in vivo.^31^

Inspired by SHAPE reagents for 2′-OH functionalization, particularly acyl imidazole chemistry, here we developed a polymerization initiator-functionalized acyl imidazole reagent, which enables covalent modification of RNA in a post-synthetic (i.e., after solid-phase synthesis or transcription) manner. Followed by the initiator integration, the subsequent atom transfer radical polymerization (ATRP) process^32−34^ resulted in the controlled growth of polymer from RNA with a narrow molecular weight distribution.

ATRP is one of the most widely used controlled radical polymerization techniques for the synthesis of polymer-biohybrids.^35−37^ In ATRP, polymerization is initiated from an alkyl halide initiator (R–X, where X can be either Cl or Br) through the reversible activation and deactivation cycles, typically mediated by a copper catalyst.^38,39^ It is important to note that we chose to incorporate the ATRP initiator and graft from the RNA rather than pre-synthesized polymers to avoid potential steric hindrance and purification issues associated with the “grafting-onto” approach. Initiating polymerization directly from the RNA allows for easier purification of the final product (separation of high-molecular-weight RNA-polymer hybrids from unreacted low-molecular-weight monomers), while expanding the range of architectural complexity by dramatically reducing steric hindrance.^14,24^

Among the various ATRP methods reported,^40^ we used the oxygen-tolerant photoinduced ATRP (photo-ATRP) technique mediated by eosin Y (EYH_2_) photocatalyst and a copper complex (X–Cu^II^/L) under green light irradiation.^41,42^ In this green-light-driven ATRP, the excited eosin Y photocatalyst transfers an electron to the X–Cu^II^/L complex, thereby (re)generating the ATRP activator (Cu^I^/L) required for polymer growth. Compared to other ATRP methods, this photoredox/Cu-catalyzed ATRP method is particularly advantageous for polymerization from nucleic acid initiators. This is due to the excellent oxygen tolerance of the dual catalysis, which allows for polymerization in low volumes (150–250 μL) without the need for deoxygenation processes such as N_2_ purging or freeze-pump-thaw cycles. These processes are typically challenging for low-volume polymerization with limited amounts of initiators and may risk mechanical degradation of the nucleic acid.^23,43^

## Results

### Development of an Acyl Imidazole Reagent for RNA Functionalization with ATRP Initiators

Compared to the other SHAPE reagents for 2′-OH modification (e.g., analogs of isatoic anhydride),^44^ acyl imidazole-based reagents are particularly useful for RNA modification because they often exhibit longer half-lives (over 30 min) with quantitative reaction yields. Thus, our synthetic strategy relied on the coupling reaction between 1,1′-carbonyldiimidazole (CDI) and β-alanine ATRP initiator (**Br-Ala**) to obtain an ATRP initiator-acyl imidazole reagent (Figure 1A). Notably, we chose a previously reported amide-based ATRP initiator (Figure 1, **Br-Ala**) due to the higher solubility of amides in aqueous buffers compared to ester-based ATRP initiators.^45,46^ The simple mixing of CDI with the **Br-Ala** in anhydrous DMSO successfully yielded the **Br-Ala**-functionalized acyl imidazole (**Br-Ala-AI**) reagent. The structure of the reagent was confirmed by ^1^H NMR spectroscopy (Figure S1). The hydrolysis kinetics of **Br-Ala-AI** under coupling conditions (20% *v*/*v* DMSO in water) was also investigated to estimate the reactivity of the reagent and determine its half-life (Figures 1C and S2). The half-life of **Br-Ala-AI** was 38 min, indicating that the reactivity of **Br-Ala-AI** was comparable to previously reported acyl imidazole reagents.^30,44^

### 2′–OH–Selective Incorporation of ATRP Initiators

**Figure 2:**
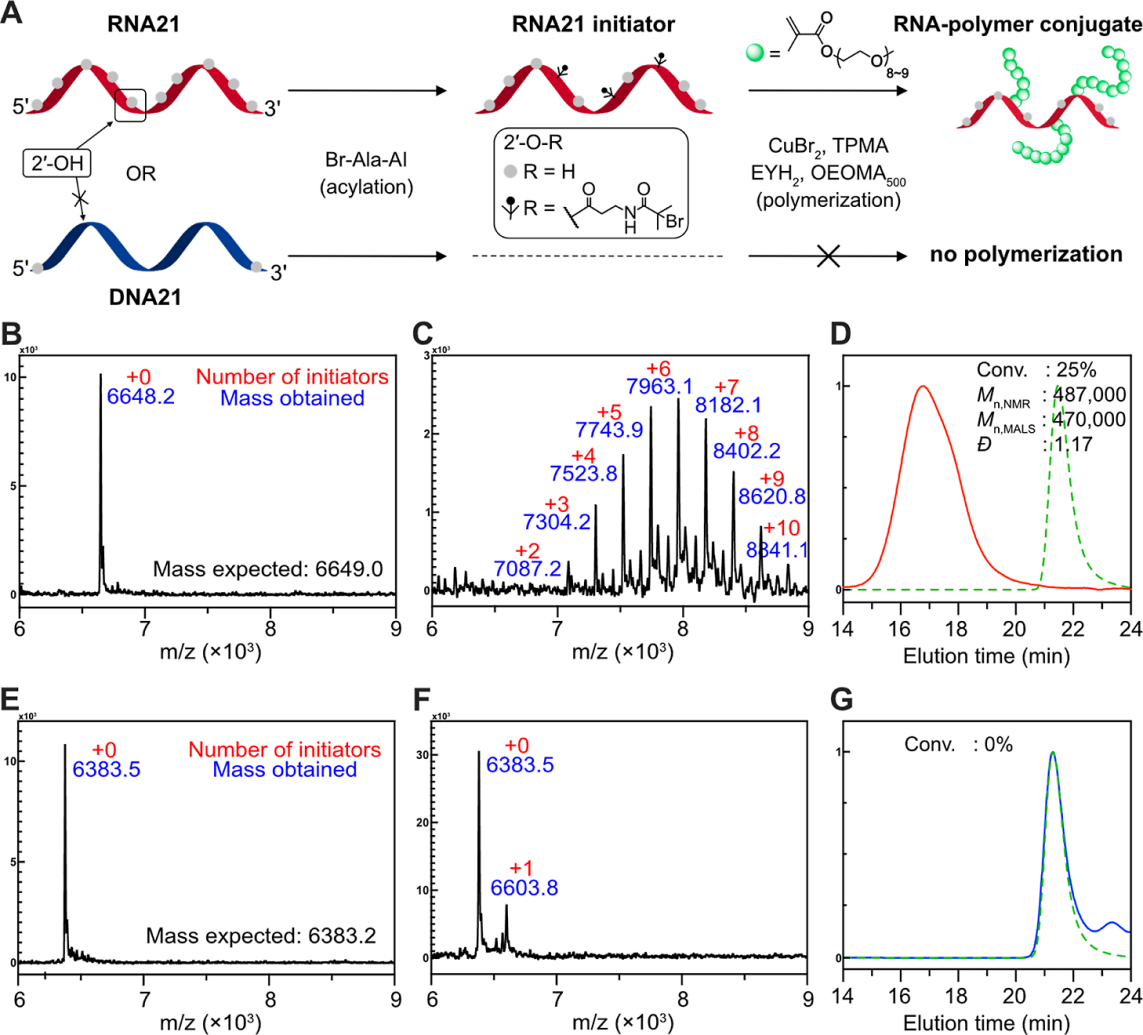
2′-OH-selective modification using **Br-Ala-AI**. (A) Schematic illustration of **Br-Ala-AI** treatment to RNA21 and DNA21 and polymerization from the oligonucleotides. (B,C) MALDI-TOF spectrum of RNA21 (B) before and (C) after **Br-Ala-AI** treatment ([M + H]^+^ region expanded for clarity). *Y*-axis is intensity (arbitrary unit), see also Figure S3. (D) SEC-MALS trace of pOEOMA_500_-grafted RNA21 (red solid line) and RNA21 initiator (green dotted line), respectively. (E,F) MALDI-TOF of DNA21 (E) before and (F) after **Br-Ala-AI** reagent treatment. (G) SEC-MALS trace of pOEOMA_500_-grafted treated DNA21 and DNA21 initiator (green dotted line). Reaction conditions: [OEOMA_500_] = 300 mM, [EYH_2_] = 0.015 mM, [CuBr_2_] = 0.9 mM, [TPMA] = 2.7 mM, [RNA21 or DNA21] = 0.075 mM under the green LEDs (520 nm, 3.7 mW/cm^2^) for 30 min at r.t., in PBS. The monomer conversion was determined by ^1^H NMR spectroscopy. PBS was used as an eluent for SEC-MALS analysis.

To test the reactivity of **Br-Ala-AI**, a 21-mer RNA (RNA21) and a 21-mer DNA (DNA21) of similar sequence (i.e., thymine instead of uracil; see Table S1 for sequences) were treated with **Br-Ala-AI** in 20% *v*/*v* DMSO in nuclease-free water (Figure 2A). After 4 h of gentle shaking at room temperature, the RNA21 or DNA21 was purified by isopropanol precipitation and three subsequent repeated centrifugation steps with a 3K molecular weight cut-off filter (MWCO filter) to remove residual unreacted and hydrolyzed **Br-Ala-AI**. Finally, the RNA21 and DNA21 strands were analyzed by mass spectrometry to determine the number of ATRP initiators attached. The comparison of the mass spectra of RNA21 before (Figure 2B) and after (Figures 2C and S3A) the treatment of **Br-Ala-AI** revealed that the peak for the initial RNA sequence at 6648.2 *m*/*z* disappeared after the reaction, indicating efficient RNA functionalization. The mass spectrum in Figure 2C with a broader mass range is shown in Figure S3A. Additionally, the normal distribution of the masses of the reaction products suggested that an average of six ATRP initiators were attached to the RNA21. In stark contrast, no dramatic change in the mass spectrum was observed when DNA21 was reacted with the **Br-Ala-AI** (Figure 2E,F). Although a small peak at 6603.8 *m*/*z* corresponding to 1 ATRP initiator attachment was observed, this is likely to be due to the coupling to the hydroxyl group at either the 5′ or 3′ terminus, consistent with a previous report.^44^ These experiments with RNA21 and DNA21 successfully demonstrated that the incorporation of ATRP initiators selectively onto 2′-OH groups in RNA is achievable. The ATRP initiators can be incorporated into post-synthetic RNA without relying on modifications added during the solid-phase RNA synthesis.

Next, we investigated the ability of the incorporated tertiary α-bromoisobutyramide moieties in RNA to initiate ATRP. We used the **Br-Ala-AI**-treated RNA21 initiator (Figure 2D) to perform the polymerization of OEOMA_500_ [i.e., oligo(ethylene oxide) methyl ether methacrylate, average *M*_n_ = 500] using EYH_2_ as the photocatalyst and CuBr_2_/TPMA [TPMA = tris(2-pyridylmethyl)amine] as the catalyst under the green light irradiation (520 nm) in phosphate-buffered saline (PBS) without deoxygenation.^41^ After the polymerization, the resulting RNA21-pOEOMA_500_ was dialyzed three times using 100K MWCO filters, passed through a Sep-Pak C18 reverse-phase cartridge to remove the photocatalyst and unreacted OEOMA_500_, and analyzed by size-exclusion chromatography equipped with multi-angle light scattering detector (SEC-MALS). Despite the low RNA21 initiator concentration of 0.075 mM, a relatively high monomer conversion of 25% and a clear shift of the SEC-MALS trace to the high molecular weight area were observed (Figure 2D). This may be attributed to the multiple initiators in a single RNA molecule (alkyl bromide = *ca.* 0.45 mM).^38^ The disappearance of the initiator peak (elution volume of *ca.* 21.4 min) indicates a nearly quantitative initiation from the RNA21 initiator, which is also confirmed by the good agreement between the absolute molecular weight obtained from SEC-MALS (*M*_n,MALS_) and the theoretical molecular weight (*M*_n,NMR_). In contrast to the reaction with RNA21 initiator, no monomer conversion was observed, when the **Br-Ala-AI**-treated DNA21 was used as an initiator (Figure 2G). The SEC-MALS peak did not shift, indicating the absence of any initiator functionalization of the DNA sequence. Presumably, the radicals generated from the residual ATRP initiator were at too low concentration and were scavenged by traces of oxygen and other impurities.

Of note, we synthesized and tested another reagent using α-bromoisobutyric acid (**BiBA-AI**), as shown in Figure S4. However, the monomer conversion with **BiBA-AI**-treated RNA21 (entry 1 in Figure S4A) was not different from that of control experiments (entries 2 and 3 in Figure S4A). This is likely to be due to the rapid hydrolysis of the **BiBA-AI** reagent in aqueous media (Figure S4B,C) caused by the short distance between the strong electron withdrawing group (i.e., Br) and acyl imidazole.^47^

## Controlled Initiator Incorporation in RNA with Helper DNA

Control over the number and the position of polymer chains in nucleic acid-polymer hybrids (e.g., multiblock copolymers, miktoarm stars, bottlebrushes, and so forth) could provide a powerful approach to tailoring their properties.^48^ With appropriately positioned polymer chains, properties such as self-assembly, mechanical strength, and therapeutic efficiency can be controlled.^19,49,50^ Therefore, we sought to control the amount of acylation to engineer the architecture of the hybrids. Kool and coworkers have shown that the use of a helper DNA that hybridizes with an RNA substrate can sequester 2′-OHs from acylation while leaving the hydroxyl groups at the predetermined positions exposed and reactive.^51^

**Figure 3:**
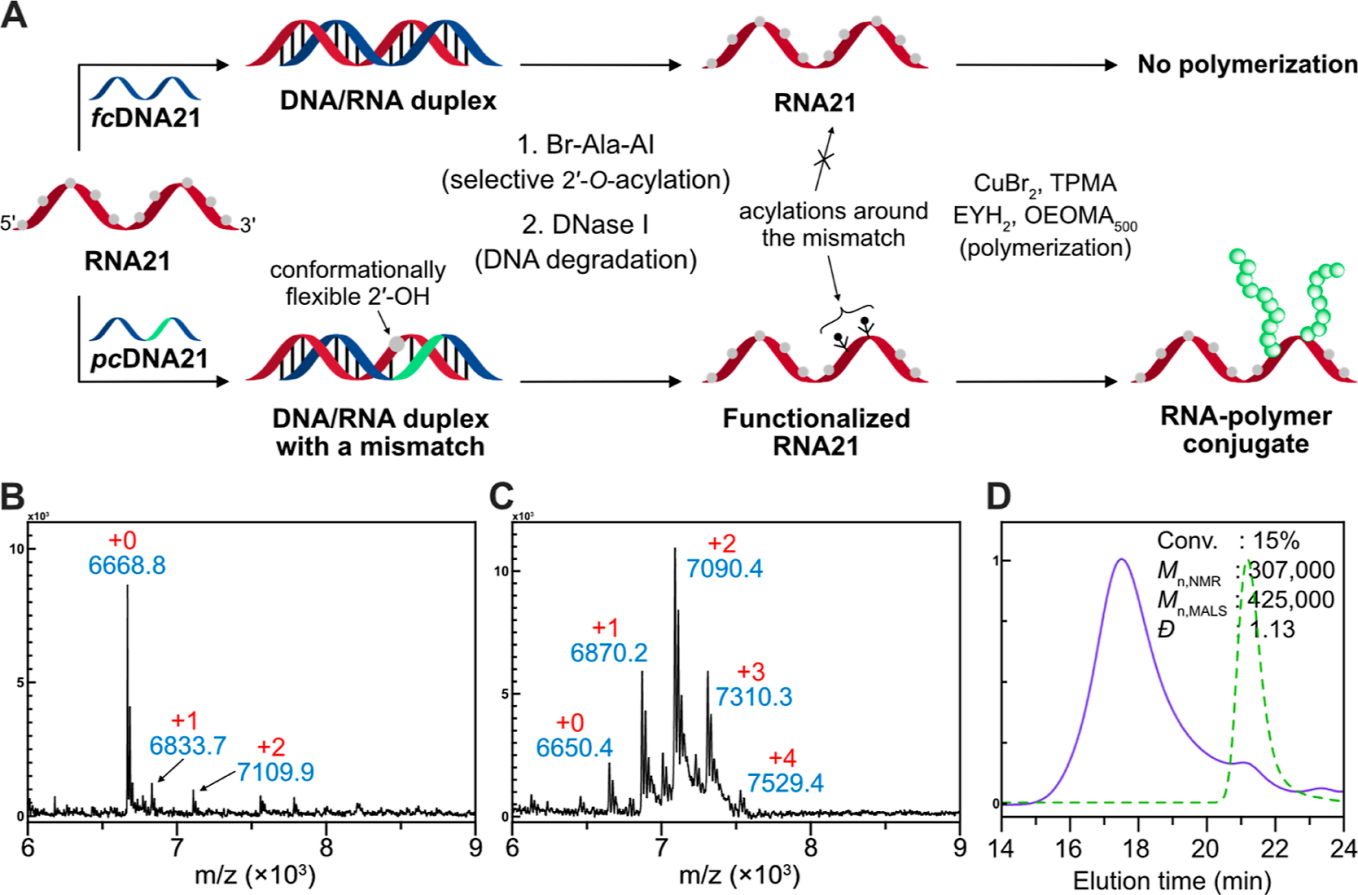
Site-selective RNA functionalization through complementary DNA-guided RNA protection. (A) Scheme of site-selective acylation on the RNA substrate (RNA21) within DNA duplex by using fully complementary helper DNA (*fc*DNA21) or partially complementary helper DNA with a mismatch in the middle (*pc*DNA21). (B,C) MALDI-TOF spectra of after **Br-Ala-AI**-treated RNA21 protected by (B) *fc*DNA21 and (C) *pc*DNA21, respectively. (D) SEC-MALS trace of pOEOMA_500_-grafted RNA21 (purple solid line) prepared by acylation at induced mismatch using *pc*DNA21 as the helper DNA. The green dotted line is a trace of the RNA21 initiator. The general polymerization condition identical as in Figure 2 is employed [(RNA oligo) = 0.075 mM (i.e., *ca.* 0.1125 mM of alkyl bromide)].

We attempted to test the control over the incorporation of the ATRP initiator groups in RNA21 by introducing fully complementary DNA (*fc*DNA21) or partially complementary DNA (*pc*DNA21) prior to **Br-Ala-AI** treatment. Once annealed, the fcDNA21/RNA21 heteroduplex is completely hybridized with 2′-OH groups inaccessible to reactions, whereas the pcDNA21 sequence annealed to SRNA21 results in a single-nucleotide mismatch in the middle of the heteroduplex (Figure 3A and Table S1). We reasoned that 2′-OH groups at the locally perturbed structure (i.e., mismatch) would be more accessible to the **Br-Ala-AI**.^52^ The annealed heteroduplex was treated with the **Br-Ala-AI** according to the previously established procedure. The reaction was conducted in MOPS buffer (pH = 7.5) for 4 h. Selective DNA degradation was then performed using DNase I.^51^ The remaining RNA product was isolated by isopropanol precipitation and filtration using a 3K MWCO filter and characterized by mass spectrometry. Interestingly, a significantly lower number of modifications in RNA21 was observed for the fcDNA21/RNA21 duplex and most of the RNA21 remained unmodified (Figure 3B). The low number of acylations in Figure 3B is likely to occur at the terminal hydroxyl groups on the 5′ and 3′ ends of the RNA.^51^ In contrast, the use of *pc*DNA21 as a helper DNA resulted in a moderate acylation yield in the range of 0 to 4 modifications (Figure 3C), which is less than compared to the result with single-stranded RNA21 (Figure 2C) and more than the result with RNA21-*fc*DNA21 duplex (Figure 3B). Previous studies have shown that unpaired nucleotides, such as loops, bulges, mismatches, and nicks, can render adjacent nucleotides thermodynamically less stable and conformationally flexible.^28,29^ Consequently, acylation around the mismatch site occurs in addition to the primary reaction site (i.e., the mismatch in our case). This can be observed through reverse transcription and subsequent polyacrylamide gel electrophoresis (PAGE).^51,52^

Next, we performed photoinduced ATRP using the RNA21 initiator prepared in the presence of *pc*DNA21 helper DNAs. As shown in Figure 3D, a monomer conversion of 15% was observed, confirming successful initiation and polymerization. Importantly, the conversion was lower than the previous result in Figure 2D (25%) due to fewer initiators within a single RNA molecule.^41,53^ Notably, the *M*_n,MALS_ was higher than the theoretical molecular weight (*M*_n,NMR_), which is likely a result of unreacted residual initiators (a peak at an elution volume of *ca.* 21.4 min). The residual initiator peak originates either from unmodified RNAs (Figure 3C) or from the lower initiation efficiency (trapping with impurities) at such a low initiator concentration.

We also tested a hairpin RNA (RNA32), which contains a double-stranded stem and a single-stranded loop in an individual RNA strand. The stem in the hairpin was able to sequester and protect some of the 2′-OHs in the RNA in a double-stranded region (Figure S5). Consistent with the results of the DNA-guided acylation, the clear shift of the mass peak of the hairpin RNA (Figure S5C,D) was inhibited when the hairpin RNA was protected by a fully complementary DNA sequence (*fc*DNA32, Figure S5E). These results indicate that control over the incorporation of ATRP initiators with selectivity within RNA sequences could be achieved by using an appropriate helper DNA. This strategy would be useful to control the density of initiators in RNA sequences toward engineering new architectures in RNA-polymer hybrids.

### Enhanced Initiator Incorporation through Water-Free Reaction

With both moderate acylation on single-stranded RNA and controlled acylation reactions on RNA/DNA duplexes to incorporate the initiators within RNA sequences, we next explored the possibility of enhancing the initiator incorporation to reach a nearly quantitative yield (i.e., 1 modification per nucleotide) that could be useful for RNA bottlebrush synthesis and engineering.^54^ Several previous studies have reported relatively high levels (25–50%) of 2′-OH functionalization by improving the water solubility of the acylating reagents, tuning the reactivity of the acylating reagents, or using nucleophilic catalysts (e.g., DMAP, 4-dimethylaminopyridine).^47,55,56^ Nonetheless, the hydrolysis of the reagents under typical reaction conditions (10–30% *v*/*v* DMSO in aqueous buffer) could prevent reaching the stoichiometric level of reaction that we desired.

**Figure 4:**
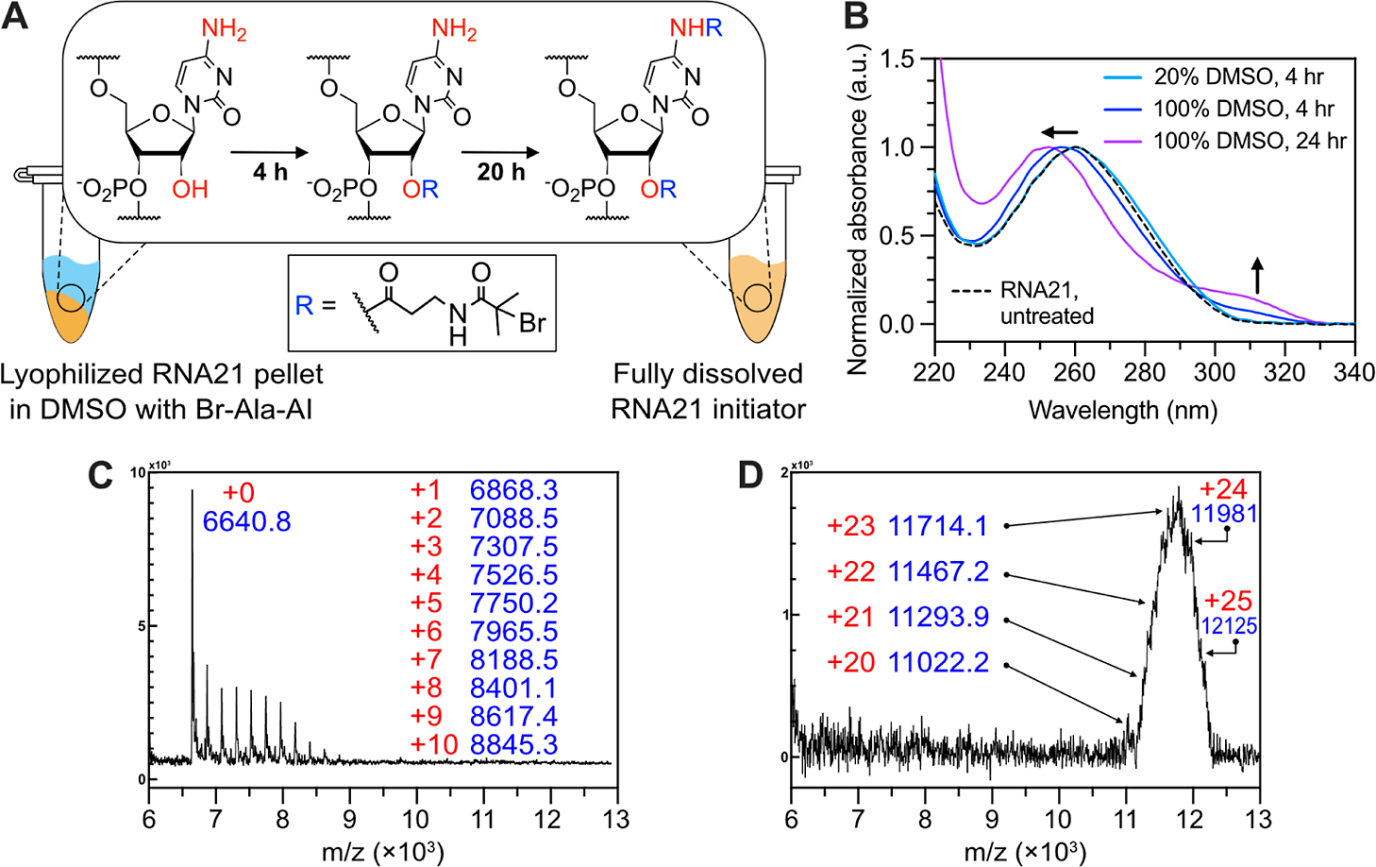
Water-free functionalization approach. (A) Schematic representation of the water-free functionalization process in 100% DMSO. (B) UV–vis spectra RNA21 after **Br-Ala-AI** treatment under different reaction conditions. (C,D) MALDI-TOF spectrum of RNA21 substrate after water-free **Br-Ala-AI** treatment for (C) 4 and (D) 24 h, respectively. See also Figure S3.

To suppress the hydrolysis of our **Br-Ala-AI** (Figure S6) and accomplish quantitative functionalization, we carried out the reaction under water-free conditions (i.e., 100% DMSO, anhydrous), as shown in Figure 4A. For this water-free functionalization reaction, RNA21 (20 nmol) was lyophilized to obtain a dry pellet and 100 μL of 0.6 M **Br-Ala-AI** in anhydrous DMSO was added to the container. Two different reaction times were tested and after 4 and 24 h of incubation with gentle shaking, the RNA products were isolated by isopropanol precipitation, and subsequential filtration was performed using a MWCO filter. We observed a significant difference between the mass spectra taken after 4 h (Figure 4C) and 24 h (Figure 4D) of the reaction. First, the shape of the mass distribution was different. As seen in Figure 4C, the peak from RNA21 at 6648.2 *m*/*z* remained as the largest peak and the intensity decreased progressively, which could be due to the stepwise coupling of the **Br-Ala-AI**: one functionalization on the single RNA nucleotide enhances the solubility of the RNA strands in DMSO that allows and enhances the next coupling reaction. In contrast, after 24 h of reaction in DMSO (Figure 4D), a normal distribution of mass peaks was observed, suggesting that the RNA strands were completely dissolved in DMSO and the **Br-Ala-AI** reacted with all the hydroxyl groups. Second, after 24 h, up to 25 modifications per 21-mer RNA were observed in the mass spectrum (Figure 4D). This excessive modification could possibly result from the excess **Br-Ala-AI** reagent reacting with the N-nucleobases under the water-free conditions.^44^

To evaluate our hypothesis, the RNA21 after the 4 or 24 h treatment with **Br-Ala-AI** was analyzed by UV–vis spectroscopy, monitoring the shift of the peak at 260 nm (A_260_), which corresponds to the absorbance of nucleobases (Figure 4B). Interestingly, after 24 h of reaction in anhydrous DMSO, a significant shift (*ca.* 7 nm) of the nucleobase absorption peak and an increase in a peak at 310 nm (A_310_) were observed, indicating an acylation reaction on the nitrogenous bases.^57^ After 4 h of reaction, the shape of the UV–vis spectrum remained largely similar to that of the untreated RNA21 (dotted black line in Figure 4B). Reactions in 20% *v*/*v* DMSO for 4 h (solid cyan line in Figure 4B) did not induce such a peak shift, because under these conditions, mainly 2′-O-adducts were formed. It is our hypothesis that at the beginning of a water-free acylation process, **Br-Ala-AI** preferentially reacts at the 2′-OH, since the amine groups in nucleobases are poorer nucleophiles than 2′-OH groups due to the electron delocalization. However, a higher number of coupling reactions to the amines on the nucleobases could be induced by extending the reaction time resulting in quantitative levels of initiator incorporation. A similar result was observed when DNA21 was used under anhydrous conditions. Despite the absence of 2′-OHs, all of the DNA21 molecules reacted with up to 8 modifications, as shown in the mass spectrum in Figure S7A. In addition, a 6 nm shift of the peak in the absorption spectrum further supports the modifications on the DNA nucleobases (Figure S7B). We performed PAGE of RNA21 and DNA21 strands. We observed the retarded migration of RNA21 (Figure S9) and DNA21 (Figure S10) after the incorporation of the ATRP initiators into the oligonucleotides.^55,56^

We performed ATRP with DNA21 or RNA21 initiators synthesized by the water-free approach. An increased monomer conversion of 65% for the RNA21 initiator (previously, 25% for the RNA21 initiator synthesized in 20% *v*/*v* DMSO in water, Figure 2D) and 9% for the DNA21 initiator (previously, 0% in Figure 2G) was observed by ^1^H NMR (Figure S8) as a result of the increased number of initiators per RNA (and DNA) in anhydrous DMSO reactions. The SEC peak of the pOEOMA500-grafted DNA21, acylated in 100% DMSO (Figure S7C), was observed in the high molecular weight region (elution volume of *ca.* 18.3 mL) as a result of polymer growth from the DNA. However, the polymerization product obtained from RNA21, synthesized in 100% DMSO, was too viscous to be injected to and analyzed by SEC. This is likely due to the accelerated termination reaction between inter- or intramolecular polymer chains induced by densely-packed initiating residues, which occurs as the conversion increases. The optimization of the polymerization conditions using sacrificial initiators could solve the problem. The results of the polymerization from oligonucleotide initiators are summarized in Table S2.

### Expanding the Substrate Scope: Grafting from Torula Yeast RNA

**Figure 5:**
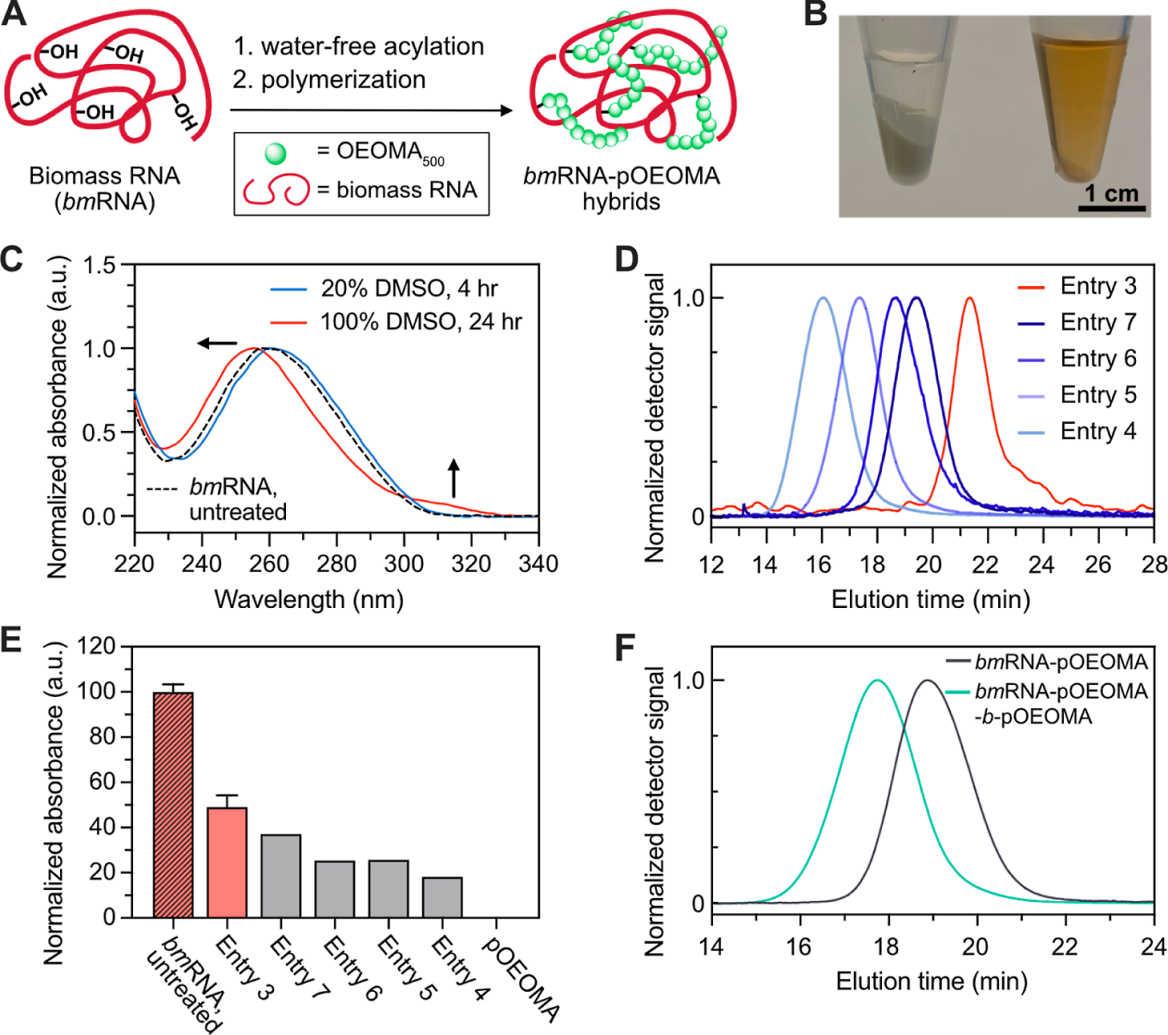
Grafting from biomass RNA. (A) Scheme of biomass RNA initiator preparation and grafting from biomass RNA initiator. (B) Biomass RNA after overnight incubation in anhydrous DMSO (left) without and (right) with **Br-Ala-AI** reagent, respectively. (C) UV–vis spectra of biomass RNA after **Br-Ala-AI** treatment under different reaction conditions. (D) A molecular weight control experiment using **Br-Ala-AI**-treated biomass RNA the as the initiator. The result of the polymer characterization by SEC-MALS is shown in Table 1. (E) Relative fluorescence intensity of pOEOMA_500_-grafted biomass RNA after staining with SYBR Gold (ex: 495 nm, em: 537 nm). (F) Chain extension experiment using biomass RNA initiator. All polymerizations were conducted under the general polymerization condition using OEOMA_500_ as the model monomer in PBS under green light irradiation for 30 min.

Over the past decade, the potential of nucleic acids from biomass as a novel building block for the fabrication of sustainable biocompatible materials has been demonstrated.^4,58,59^ This inspired us to explore our method and extend the scope of RNA that can be used to biomass RNA (*bm*RNA) extracted from torula yeast (Figure 5A). We first prepared a *bm*RNA macroinitiator by mixing 10 mg of *bm*RNA with 100 μL of 0.6 M **Br-Ala-AI** reagent in anhydrous DMSO. After overnight incubation under anhydrous conditions with gentle shaking, we observed almost complete dissolution of the *bm*RNA in DMSO, suggesting successful modifications (Figure 5B, right). When *bm*RNA was incubated in the DMSO without **Br-Ala-AI** (Figure 5B, left), the RNA remained mostly undissolved due to the poor solubility of native RNA in organic solvents. To isolate the functionalized bmRNA (in the supernatant) and to remove the unreacted bmRNA (in a pellet form), the mixture was centrifuged and the supernatant was collected. To precipitate the bmRNA initiator from the supernatant, sodium acetate and isopropanol were added. The resulting precipitate containing the bmRNA initiator was collected by centrifugation, and if necessary, a 3K MWCO filter was used to further purify the sample. Finally, the bmRNA initiator was characterized by UV–vis spectroscopy (Figure 5C). A change in the spectrum was observed after water-free functionalization (Figure 5C, solid red line), similar to the results obtained with RNA21 as a result of modifications to the nucleobases. Of note, the change in the shape of the UV–vis spectrum of *bm*RNA (Figure 5C) after water-free acylation was less significant compared to the RNA oligomer (RNA21, Figure 4B). This is presumably because of the aggregation of the *bm*RNA strands in DMSO and the use of the higher amount of RNA substrate (15 and 0.42 μmol of ribonucleotides for *bm*RNA and RNA21, respectively). Using ^1^H NMR spectroscopy, the ratio of ribonucleotides to ATRP initiator residues incorporated into the bmRNA was estimated to be 4.25 acylations per 10 ribonucleotides (Figure S11).

Next, we examined the capability of the *bm*RNA macroinitiator prepared by the water-free approach to initiate the photo-ATRP of OEOMA_500_ monomer. As shown in Table 1, negligible monomer conversion was observed in negative control experiments lacking 2′-OH in the substrate (Table 1, entry 1), **Br-Ala-AI** treatment (Table 1, entry 2), or green-light for polymerization (Table 1, entry 3). In contrast, a high monomer conversion (>50%) was observed only when **Br-Ala-AI**-treated *bm*RNA was used as the macroinitiator under green light irradiation. In addition, the conversion gradually increased as more initiators were used for polymerization (Table 1, entries 4–7), which is consistent with other polymerization results in photo-ATRP.^41,53^ The polymerization products (*bm*RNA-pOEOMA) were isolated by using 100K MWCO filter and a Sep-Pak cartridge as mentioned above and then analyzed by SEC-MALS. The shift of the monomodal SEC-MALS traces in Figure 5D demonstrated that the molecular weights of the polymer grafts from *bm*RNA were controlled by simply changing the concentration of the *bm*RNA macroinitiator. SEC-MALS analysis of the *bm*RNA macroinitiator showed a relatively low dispersity (*D̵*) of 1.15 with a number average molecular weight (*M*_n,MALS_) of 12.1 kDa (Table 1, entry 3). This is probably due to the lower number of modifications and the precipitation of short *bm*RNA strands during the anhydrous acylation process.

**Table 1:** Results of Grafting from Biomass Nucleic Acids^a^

entry	initiator	nucleic acid concentration (mg/mL)	conv^b^ (%)	*M*_n,MALS_ (kDA)	*D̵*	degree of polymerization^c^
1	biomass DNA	0.5	3	N/A	N/A	N/A
2^d^	biomass RNA	0.5	1	N/A	N/A	N/A
3^e^	biomass RNA	0.5	1	12.1	1.15	*bm*RNA initiator
4	biomass RNA	0.5	57	1238.4	1.41	2452
5	biomass RNA	1.5	60	496.7	1.37	969
6	biomass RNA	4.5	65	239.6	1.22	455
7	biomass RNA	9.0	68	132.7	1.24	241

a SEC-MALS traces are shown in Figure 5D. Biomass DNA extracted from salmon was used as the initiator for entry 1 after **Br-Ala-AI** treatment in anhydrous DMSO. Reaction conditions: [OEOMA_500_] = 300 mM, [EYH_2_] = 0.015 mM, [CuBr_2_] = 0.9 mM, [TPMA] = 2.7 mM, [*bm*RNA] = 0.5–9.0 mg/mL under the green LEDs (520 nm, 3.7 mW/cm^2^) for 30 min at r.t., in PBS.

b The conversion was determined by ^1^H NMR spectroscopy (Figure S13).

c The degree of polymerization for the chains grafted from *bm*RNA initiator was calculated using the following equation: [*M*_n,MALS_ of the polymer – *M*_n,MALS_ of *bm*RNA initiator (12.1 kDA, entry 3)] divided by 500, the molar mass of OEOMA_500_.

d *bm*RNA, without treatment with **Br-Ala-AI** was used.

e The reaction was performed in the dark, without green-light irradiation.

To determine the presence of RNA in *bm*RNA-pOEOMA conjugates, we stained the polymerization products with the RNA-staining fluorogenic dye (SYBR Gold). This cyanine-based dye exhibits high fluorescence enhancement upon binding to nucleic acids. We used SYBR Gold with untreated *bm*RNA, *bm*RNA macroinitiator, and *bm*RNA-pOEOMA hybrids from Table 1 dissolved in water at a final concentration of 1 mg/mL. We treated 100 μL of each sample with SYBR Gold and recorded the fluorescence intensity in a microplate reader (Figure 5E). As expected, strong fluorescence enhancement by SYBR Gold was observed for all *bm*RNA macroinitiators and *bm*RNA-pOEOMA hybrids. In contrast, no fluorescence was observed for pOEOMA initiated from a conventional ATRP initiator (i.e., HEBiB, 2-hydroxyethyl α-bromoisobutyrate). Moreover, we also observed the lower fluorescence intensity of the *bm*RNA macroinitiator or polymer-functionalized biomass RNA (Figure 5E, entries 3–7) compared to the untreated *bm*RNA. This is possibly due to the inhibited intercalation of the SYBR Gold dye molecules between the planar nucleobases.^60,61^

One of the important features of controlled radical polymerization^62^ is the living polymer chain end, which facilitates the synthesis of di- or multi-block copolymers.^63^ To investigate the fidelity of the chain end grafted from the *bm*RNA macroinitiator, we conducted a chain extension experiment (Figure 5F). Initially, *bm*RNA-pOEOMA was synthesized under the general polymerization conditions (*M*_n,MALS_ = 146.7 kDa, conv. = 66%). An aliquot of the polymerization product was taken without further purification and combined with the ATRP catalytic mixture for the second polymerization.^46,53^ After 30 min of the second polymerization reaction under green light, 49% of monomer conversion was observed with a clear shifting of the SEC-MALS trace to the high molecular weight values indicating preserved chain end activity after the first polymerization (Table S3).

### Fabrication of *bm*RNA-pOEOMA Hydrogel and *bm*RNA-pNIPAM Hybrids

**Figure 6:**
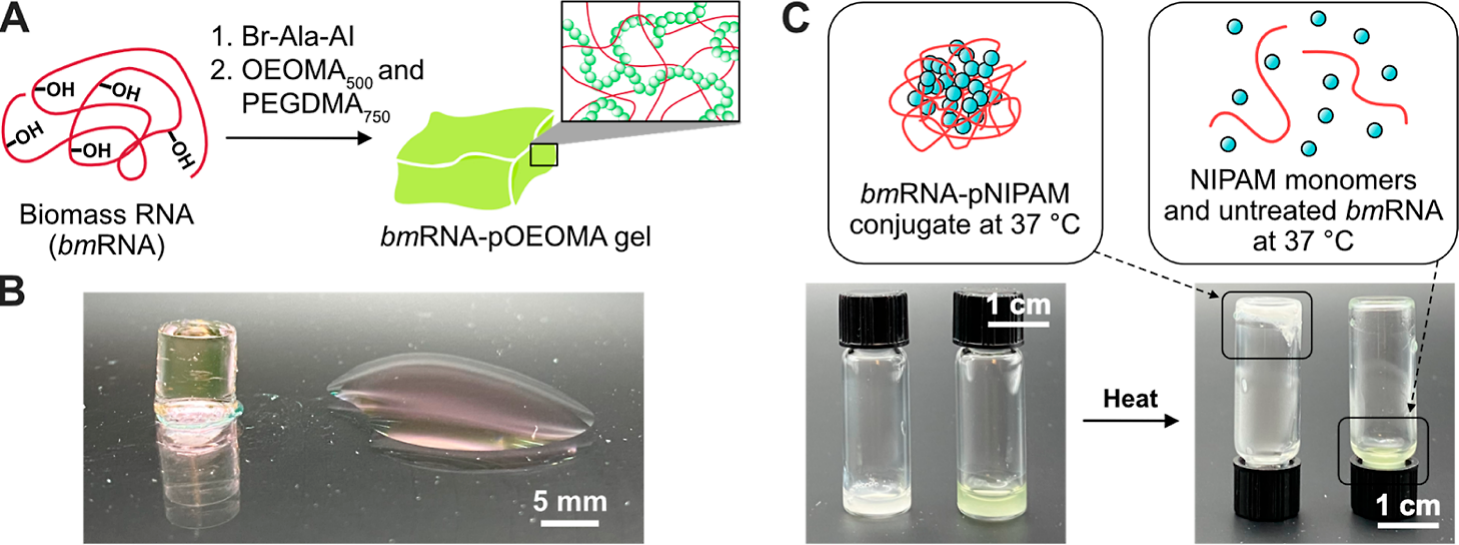
(A) Scheme of *bm*RNA-pOEOMA hydrogel synthesis by copolymerization of OEOMA_500_ and PEGDMA_750_ from *bm*RNA macroinitiator. (B) Image of *bm*RNA-pOEOMA hydrogel after 30 min of polymerization using *bm*RNA initiator (final concentration of 0.5 mg/mL) under the general polymerization condition in the presence of PEGDMA_750_ (final concentration of 60 mM). (left) *bm*RNA macroinitiator synthesized under water-free conditions was used as the initiator. (right) Unmodified biomass RNA without **Br-Ala-AI** treatment was used. The light pink color is due to eosin Y photocatalyst. (C) Images after polymerization of NIPAM with biomass RNA (left) with and (right) without **Br-Ala-AI** treatment, respectively. Reaction condition: [NIPAM]/[EYH_2_]/[CuBr_2_]/[Me_6_TREN] = 1000/0.045/2.7/8.1, [*bm*RNA] = 0.5 mg/mL and [NIPAM] = 1000 mM under green light irradiation (520 nm, 3.7 mW cm^–2^) for 30 min, in 80% *v*/*v* DMSO in PBS. Me_6_TREN = tris[2-(dimethylamino)ethyl]amine.

We applied the functionalization of *bm*RNA and subsequent polymerization to the fabrication of macroscopic structures. As shown in Figure 6A, we synthesized a hydrogel by the photo-ATRP of OEOMA_500_ using the *bm*RNA macroinitiator in the presence of a crosslinker [PEGDMA_750_, poly(ethylene glycol) dimethacrylate, average *M*_n_ = 750]. The polymerization reaction was performed in a 96-well plate using a photoreactor (520 nm). As a result of polymerization, a macroscopic hydrogel was obtained, as shown in Figure 6B. When *bm*RNA without **Br-Ala-AI** treatment was used instead of *bm*RNA macroinitiator, no polymerization occurred (Figure 6B, right), indicating negligible polymerization initiation induced by the photredox/Cu dual catalysis.

Taking advantage of the increased solubility of ATRP initiator-functionalized RNA in DMSO, we pursued polymerization from the *bm*RNA macroinitiator in an organic solvent (i.e., DMSO). We reasoned that this would be a promising new approach for the fabrication of nucleic acid-polymer amphiphiles.^64−66^ In addition to water-soluble polymers, such as polyethylene glycol (PEG), that provide degradation resistance, other polymers such as poly(*N*-isopropylacrylamide) (pNIPAM) can provide temperature-responsiveness to RNA-based biomaterials. Using *N*-isopropylacrylamide (NIPAM) as the model monomer, we performed the polymerization reactions in 80% *v*/*v* DMSO in PBS. The resulting *bm*RNA-pNIPAM hybrid was dialyzed in ice-cold water and transferred to a 0.5-dram vial. As shown in Figure 6C, when the temperature was increased above the lower critical solution temperature (LCST) of pNIPAM (Figure S12A), the *bm*RNA-pNIPAM hybrids aggregated and became a gel. In contrast, when *bm*RNA without **Br-Ala-AI** treatment was used instead of the *bm*RNA macroinitiator, no polymerization of NIPAM was observed and the solution remained as a flowable liquid even after heating (Figure 6C). Shrinkage of *bm*RNA-pNIPAM was also observed by dynamic light scattering (Figure S12B). These results demonstrate that novel materials can be fabricated from *bm*RNA, functionalized using our ATRP initiator reagent with a wide range of monomers under anhydrous polymerization conditions.

## Conclusions

We have developed a universal reagent for the covalent functionalization of RNA with one or more ATRP initiators. Up to 10 modifications of the 2′-OHs in a 21-mer RNA were achieved under general acylating conditions (i.e., 20% *v*/*v* DMSO in water), which can be further engineered with the aid of a suitable helper DNA or by performing coupling reactions in the hydrolysis-limited environment (i.e., anhydrous DMSO). We have shown that we can extend the scope of substrates from short synthetic RNA oligonucleotides to RNA sequences extracted from biomass. Treatment of the *bm*RNA with the **Br-Ala-AI** and subsequent polymerization demonstrated the controllability over the molecular weights of the pOEOMA_500_ grafts in addition to the living polymerization behavior. Synthesis of *bm*RNA-pOEOMA hydrogel and temperature-responsive *bm*RNA-pNIPAM hybrids was also demonstrated in water and DMSO, respectively. Our novel and versatile approach to RNA-polymer hybrids circumvents the challenges of other coupling methods, including azide–alkyne cycloadditions, electrostatic interactions, or hydrogen bonding. We anticipate that the direct and universal incorporation of acyl imidazole-based ATRP initiators and subsequent polymerization will expand the scope of RNA substrates for the combination with polymers and increase their utility as innovative biomaterials. In addition, the high coupling yield and, thereby, improved solubility of RNA initiators in the organic-phase will vastly broaden the choice of polymerizable monomers that would be interesting for nucleic acid therapeutics^67^ or pathogen detection.^68^

## Supporting Information Available

Additional experimental details and information including ^1^H NMR, additional mass spectrum, additional GPC results, mass spectra, gel electrophoresis results, monitoring of LCST transition, DLS, and table for the summary of reaction conditions and results (PDF)
