# Supplementary material for: RNA-Polymer Hybrids via Direct and Site-Selective Acylation with the ATRP Initiator and Photoinduced Polymerization
Source: J Am Chem Soc. 2023 Jun 26;145(26):14435–45. doi: 10.1021/jacs.3c03757 (PMC10326879; doi:10.1021/jacs.3c03757)
Supplement: Supplementary file 1 — ja3c03757_si_001.pdf [file ja3c03757_si_001.pdf]

Supporting information

RNA-Polymer Hybrids via Direct and Site-Selective Acylation with ATRP Initiator and Photoinduced Polymerization

Jaepil Jeong,[1,2] Xiaolei Hu,[1] Hironobu Murata,[1] Grzegorz Szczepaniak,[1,3] Marta Rachwalak,[1,2] Anna Kietrys,[1,2] Subha R. Das,[1,2*] and Krzysztof Matyjaszewski,[1*]

[1]Department of Chemistry, Carnegie Mellon University, Pittsburgh, Pennsylvania 15213, United States.

[2]Center for Nucleic Acids Science & Technology, Carnegie Mellon University, Pittsburgh, Pennsylvania 15213, United States

[3]University of Warsaw, Faculty of Chemistry, Pasteura 1, 02-093 Warsaw, Poland

*Correspondence: S.R.D (srdas@andrew.cmu.edu); K.M. (km3b@andrew.cmu.edu)

Table of Contents

Experimental details S3

Materials S3

Instruments S3

Procedures S4

Synthesis of Br-Ala-AI reagent S4

General procedure for oligonucleotide functionalization with Br-Ala-AI S4

General procedure for EY/Cu-catalyzed photo-ATRP S4

Controlled initiator incorporation in RNA with helper DNA S5

Procedure for the water-free RNA functionalization S5

Chain extension experiment using biomass RNA (*bm*RNA) macroinitiator S6

Procedure for the fabrication of *bm*RNA-pOEOMA hydrogel in 96 well plate S6

Grafting NIPAM from *bm*RNA macroinitiator S7

Supplementary Data S8

Reference S26

Experimental details

Materials

Unless otherwise stated, all the chemicals, including torula yeast RNA (type VI) were purchased from Sigma Aldrich. Molecular weight cut-off (MWCO) filters (Amicon ultra centrifugal filter) were purchased from Sigma Aldrich. All the oligonucleotides used in this study were purchased from IDT. Tris(2-pyridylmethyl)amine (TPMA) was purchased from AmBeed. Carbonyldiimidazole (CDI) was purchased from TCI America. All the organic solvents, 40% PAGE gel mix, 10X phosphate-buffered saline (PBS), and 10X tris-borate-EDTA buffer were purchased from Fisher Scientific. SYBR Gold dye (10000X in DMSO) was purchased from Invitrogen. Sep-Pak C18 cartridge was purchased from Waters. DNase I was purchased from New England BioLabs. 96 well plate was purchased from Greiner (CellStar ®). Me_6TREN (*N*,*N*,*N*´,*N*´´,*N*´´- tris[2-(dimethylamino)ethyl]amine) was received from Koei Chemical Co., Ltd.

Instruments

^{1}H NMR spectra were recorded by Bruker Avance III 500 MHz spectrometer. NanoDrop One UV-Vis spectrophotometer (ThermoFisher Scientific) was used to obtain the absorbance spectrum. Polyacrylamide gel after PAGE was imaged by Typhoon FLA 9000 gel scanner (GE Healthcare Life Sciences). UltrafleXtreme MALDI-TOF Mass Spectrometer (Bruker) was used for the characterization of oligonucleotides using MTP 384 Target Plate Ground Steel (Bruker). The fluorescence intensity of SYBR Gold was measured by using Infinite® M1000 (Tecan) microplate reader. The green LED strip for polymerization was purchased from aspectLED and the strip was mounted inside a glass jar (height = 7 cm, diameter = 9 cm). Fabrication of biomass RNA gel was carried out using Lumidox II 96-Well LED Arrays (λ = 540 nm) and Lumidox II Controller. Zetasizer Nano ZS (Malvern) was used for dynamic light scattering analysis. UV−VIS spectrometer (Lambda 2, PerkinElmer) was used for the determination of LCST of RNA-pNIPAM conjugate.

Procedures

Synthesis of Br-Ala-AI reagent

β-Alanine initiator (**Br-Ala**, *N*-α-bromoisobutyryl-β-alanine) was synthesized by following the previously reported procedure.[1] Next, 1.2 M CDI stock and 1.2 M **Br-Ala** initiator were prepared in the separate Eppendorf tube by dissolving 194.58 mg of CDI and 285.7 mg of **Br-Ala** in anhydrous DMSO at the final volume of 1 mL, respectively. Finally, equal volumes of 1.2 M CDI stock and 1.2 M **Br-Ala** were mixed and incubated for 10 min at room temperature under gentle shaking. **Br-Ala**-functionalized acyl imidazole reagent (**Br-Ala-AI**) at 0.6 M concentration in anhydrous DMSO was stored in a − 20 °C freezer until further use.

General procedure for oligonucleotide functionalization with Br-Ala-AI

30 nmol of oligonucleotide substrate and 150 μL of 0.6 M **Br-Ala-AI** were mixed and the volume was brought to 750 μL by adding water (final DMSO concentration is 20% *v*/*v*). The mixture was thoroughly mixed, followed by incubation for 4 h at room temperature under gentle shaking. Next, functionalized oligonucleotide was precipitated by using sodium acetate and 1.5X volume of isopropanol at -80℃ for overnight. The precipitates were collected by centrifugation (13000g, 30 min) at 4℃. The pellet was redissolved in 500 μL of nuclease-free water. Then, the solution was further purified by using 3K MWCO filter (Amicon ultra centrifugal filter) for three repeating centrifugations. After the purification step using the MWCO filter, the solution remaining in the filter was collected and the concentration of oligonucleotide was determined by measuring A_{260} and the appropriate extinction coefficient calculated by Oligo Analyzer (IDT). Mass spectra of the oligonucleotides were recorded by using 3-Hydroxypicolinic acid dissolved in 50% acetonitrile in water containing 10 mg/mL diammonium hydrogen citrate as a matrix for MALDI-TOF.

General procedure for EY/Cu-catalyzed photo-ATRP

Prior to polymerization, stock solutions of reagents were prepared as follows:

$CuBr_2$ stock	:	15 mg of $CuBr_2$ in 1194 μL of 50% *v*/*v* DMSO in water
TPMA stock	:	15 mg of TPMA in 153.6 μL of DMSO
EYH_2 stock	:	9.72 mg of EYH_2 in 10 mL of DMSO

$OEOMA_{500}$ stock : 250 mg of $OEOMA_{500}$ in 1 mL of water

Next, 150 µL of $OEOMA_{500}$ stock, 4 µL of $CuBr_2$ stock, 2 µL of TPMA stock, 2.5 µL of EYH_2 stock, 25 µL of 10X PBS and oligonucleotide initiator (final concentration of 75 µM) was mixed, and the final volume of the mixture was brought to 250 µL by adding water. The reaction cocktail was transferred into a 250 µL glass insert followed by irradiation of green light (λ = 520 nm, 3.7 mW cm^{-2}) at room temperature to initiate polymerization. The final concentrations of reagents were as follows: [$OEOMA_{500}$] = 300 mM, [$CuBr_2$] = 0.9 mM, [TPMA] = 2.7 mM, and [EYH_2] = 0.015 mM. After 30 min of polymerization under green light, 50 µL of the reaction mixture was used for ^{1}H NMR analysis to determine monomer conversion. The rest of the mixture was used for the determination of absolute molecular weight ($M_{n,MALS}$) using size-exclusion chromatography equipped with a multi-angle light scattering detector (SEC-MALS). PBS was used as an eluent for SEC-MALS.

Controlled initiator incorporation in RNA with helper DNA

20 nmol of RNA substrate and 32 nmol of corresponding helper DNA were mixed in a hybridization buffer (150 mM NaCl, 100 mM MOPS, 10 mM $MgCl_2$). The mixture was annealed by heating at 95℃ for 8 min in a heat block and slowly cooling down to room temperature at the rate of approximately 0.5℃/min. Following the annealing process, 0.6 M **Br-Ala-AI** (final concentration of 0.12 M) and 0.5 M MOPS buffer (pH 7.5) containing 50 mM $MgCl_2$ and 0.5 M NaCl was added at the final concentration of 100 mM of MOPS buffer. The mixture was incubated for 4 h at room temperature under gentle shaking. To the incubated mixture was added 5 µL of DNase I (10 units) and 12 µL of 10X DNase I reaction buffer (100 mM Tris-HCl, 25 mM $MgCl_2$, and 5 mM $CaCl_2$, pH 7.6). The mixture was incubated at 37℃ for 1 h. After the incubation, the functionalized RNA was purified by using 3K MWCO filters as presented above and the concentration of RNA was calculated by measuring A_{260} and using Beer-Lambert Law.

Procedure for the water-free RNA functionalization

100 µL of 0.6 M **Br-Ala-AI** in DMSO were mixed with 20 nmol of lyophilized RNA pellet or 10 mg of biomass RNA (*ca.* 15 µmoles). After 24 hours of incubation at room temperature, functionalized RNA strands were purified by isopropanol precipitation

and subsequent dialysis using MWCO filters as presented above. Finally, the absorbance maximum of the nucleobases in the range of 250–260 nm was measured to calculate the concentration of RNA using Beer-Lambert Law.

Chain extension experiment using biomass RNA (*bm*RNA) macroinitiator

150 μL of $OEOMA_{500}$ stock, 4 μL of $CuBr_2$ stock, 2 μL of TPMA stock, 2.5 μL of EYH_2 stock, 25 μL of 10X PBS and *bm*RNA macroinitiator (final concentration of 6 mg/mL) was mixed, and the final volume of the mixture was brought to 250 μL by adding water. The ATRP "cocktail" was transferred into a 250 μL glass followed by irradiation of green light (λ = 520 nm, 3.7 mW cm^{-2}) at room temperature to initiate polymerization. After 30 min of polymerization under green light, 125 μL of the reaction mixture was used for analysis using ^1H NMR spectroscopy (to determine monomer conversion, $conversion_{1st}$) and SEC-MALS. To the rest of the mixture was added 75 μL of $OEOMA_{500}$ stock, 2 μL of $CuBr_2$ stock, 1 μL of TPMA stock, 1.25 μL of EYH_2 stock, 12.5 μL of 10X PBS and 30.4 μL of water. The mixture was thoroughly mixed and transferred into a 250 μL glass followed by irradiation of green light to restart polymerization. After 30 min of reaction, 50 μL of the mixture was used for ^1H NMR analysis to determine monomer conversion ($conversion_{2nd}$). The rest of the mixture was used for SEC-MALS analysis. The actual monomer conversion of the 2nd polymerization ($conversion_{2nd,actual}$) was determined by the equation presented below.

$$Conversion_{2nd,actual} = Conversion_{2nd} - \frac{Conversion_{1st}}{2}$$

Procedure for the fabrication of *bm*RNA-pOEOMA hydrogel in 96 well plate

Prior to polymerization, $PEGDMA_{750}$ stock was prepared by dissolving 450 mg of $PEGDMA_{750}$ in 1 mL of water. Next, 150 μL of $OEOMA_{500}$ stock, 4 μL of $CuBr_2$ stock, 2 μL of TPMA stock, 2.5 μL of EYH_2 stock, 25 μL of 10X PBS, 25 μL of $PEGDMA_{750}$ stock and *bm*RNA macroinitiator (final concentration of 0.5 mg/mL) was mixed, and the final volume of the mixture was brought to 250 μL by adding water. The ATRP "cocktail" was transferred into a 96-well plate followed by irradiation of green light (λ = 540 nm, 20 mW cm^{-2}) at room temperature. After 30 min of polymerization, the LED light was turned off to stop the reaction.

Grafting NIPAM from *bm*RNA macroinitiator

Prior to polymerization, NIPAM stock was prepared by dissolving 339.5 mg of NIPAM in DMSO at a final volume of 1 mL. Next, 83 µL of NIPAM stock, 12 µL of $CuBr_2$ stock, 6 µL of TPMA stock, 7.5 µL of EYH_2 stock, 10 µL of DMF, 25 µL of water, 25 µL of 10X PBS and *bm*RNA macroinitiator stock in DMSO was mixed (final concentration of 0.5 mg/mL), and the final volume of the mixture was brought to 250 µL by adding DMSO. The ATRP "cocktail" was transferred into a 250 µL glass insert followed by irradiation of green light (λ = 520 nm, 3.7 mW cm^{-2}) at room temperature to initiate polymerization. The final concentrations of reagents were as follows: [NIPAM] = 1000 mM, [$CuBr_2$] = 2.7 mM, [TPMA] = 8.1 mM, and [EYH_2] = 0.045 mM. After 30 min of polymerization under green light, polymerization was stopped by turning the light off.

Supplementary Data

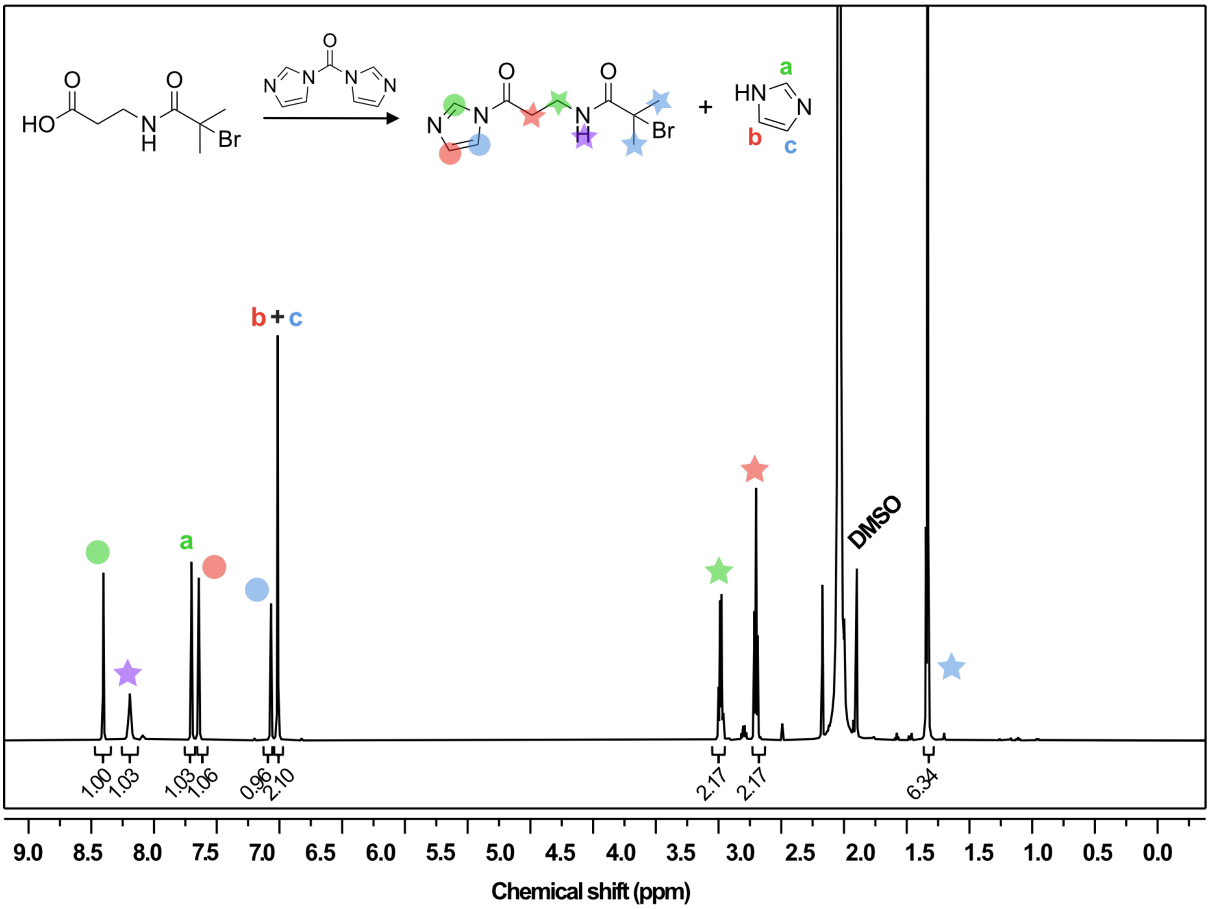

Figure S1. ^{1}H NMR of **Br-Ala-AI**. ^{1}H NMR (500 MHz, DMSO-d_6) δ 8.40 (s, 1H), 8.19 (t, *J* = 5.3 Hz, 1H), 7.70 (t, *J* = 1.5 Hz, 1H), 7.65 (s, 1H), 7.07 (d, *J* = 1.6 Hz, 1H), 7.01 (d, *J* = 0.9 Hz, 2H), 3.48 (q, *J* = 6.3 Hz, 2H), 3.20 (t, *J* = 6.5 Hz, 2H), 1.83 (s, 6H).

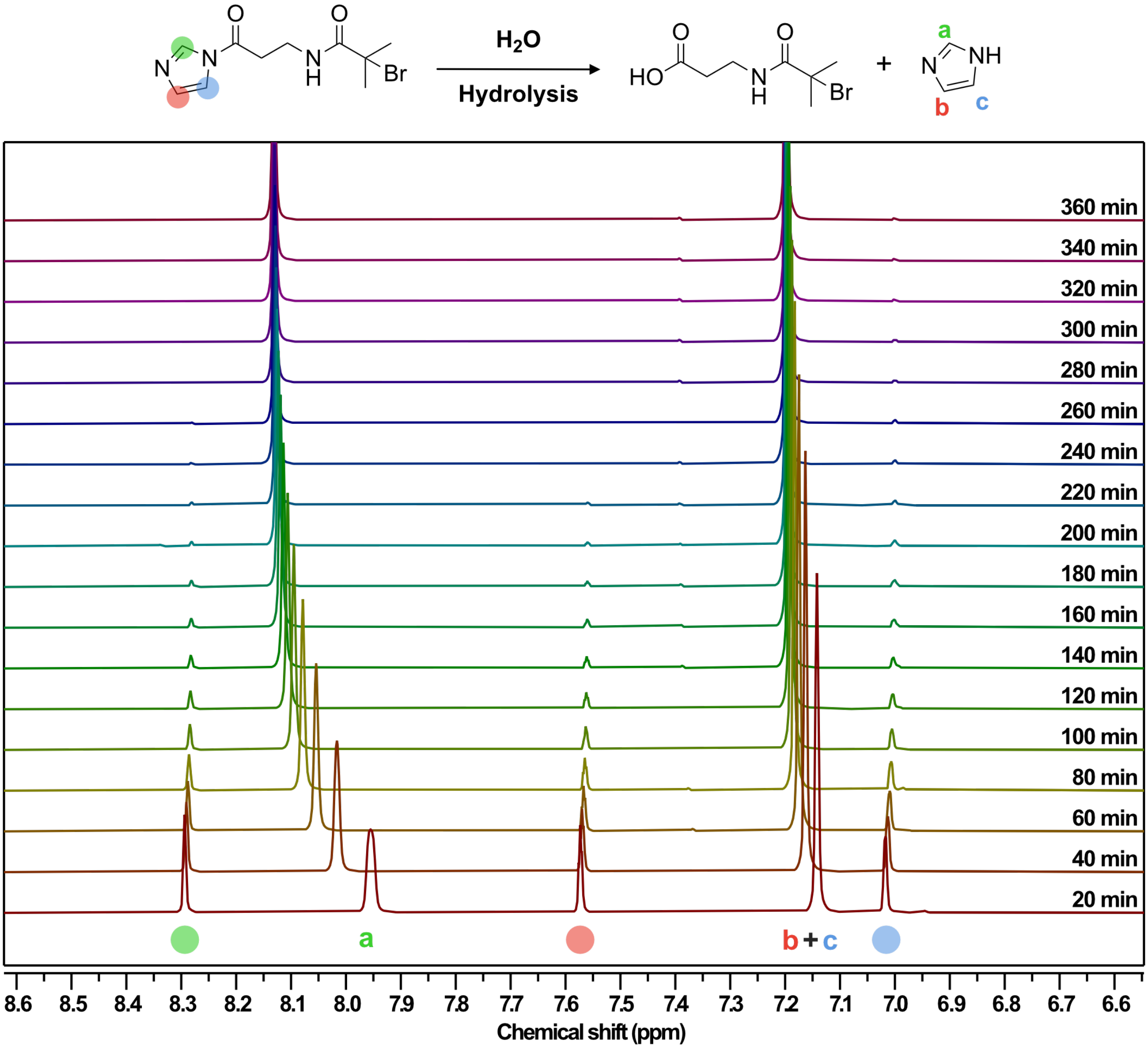

Figure S2. Hydrolysis of **Br-Ala-AI** in 20% *v*/*v* DMSO in water. ^{1}H NMR spectra of **Br-Ala-AI** in 20% *v*/*v* DMSO in water at various time points.

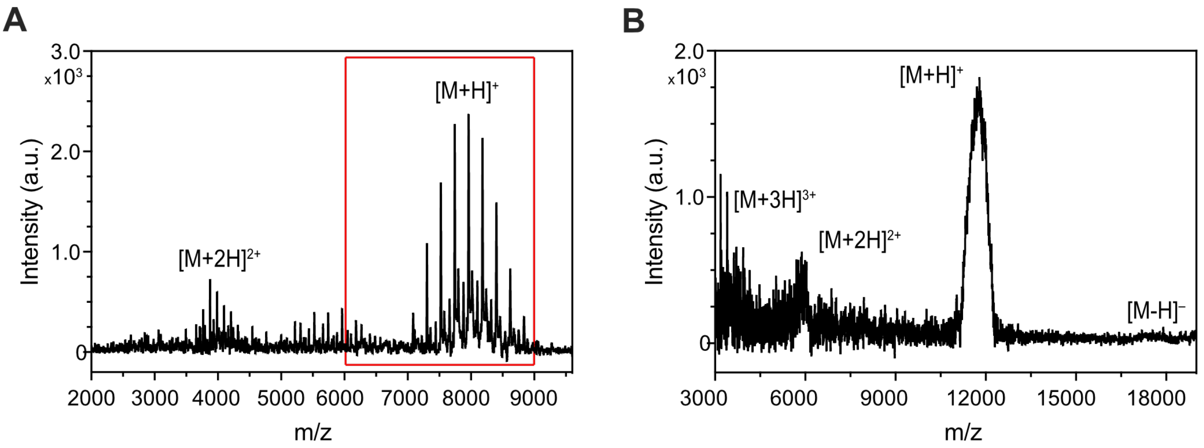

Figure S3. (A) Full MALDI-TOF spectrum shown in Figure 2C (red box). RNA21 after **Br-Ala-AI** treatment was the substrate for the mass analysis. (B) MALDI-TOF spectrum shown in Figure 4D at a broader mass range. RNA21 after water-free **Br-Ala-AI** treatment for 24 h was the substrate for the mass analysis. Each 2'-*O*-acylation with **Br-Ala-AI** results in an increase of 219–220 mass units. In Fig 2C, all peaks with noted mass obtained are within 4–6 mass units in the ionized RNA species.

A

Entry	initiator	Conversion (%)
1	BiBA-AI-treated RNA21	2
2	BiBA-AI-treated DNA21	2
3	RNA21, untreated	3

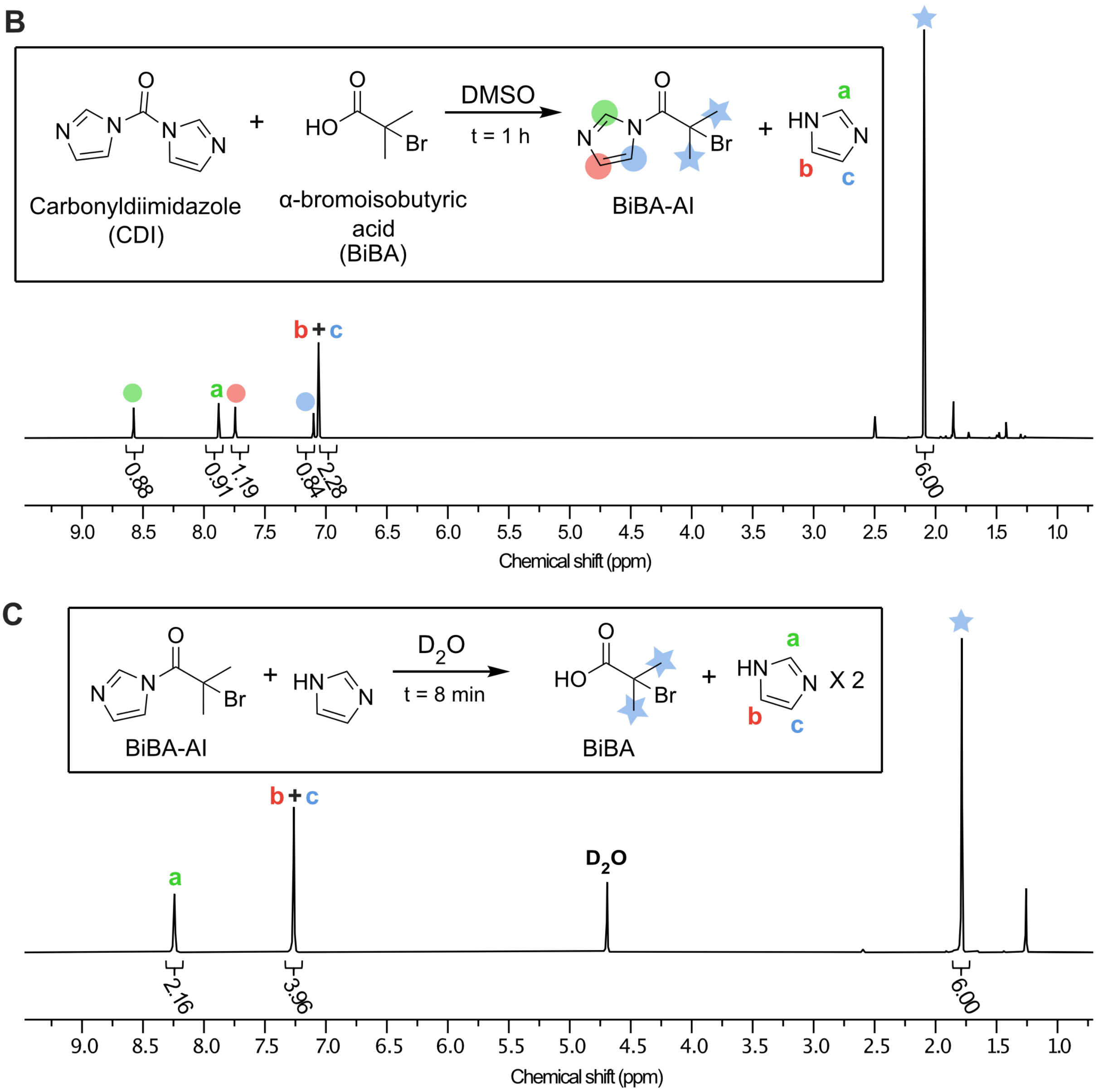

Figure S4. Use of **BiBA-AI** for RNA functionalization and polymerization. (A) Table for the polymerization result using **BiBA-AI**-treated RNA as the initiator. The sequences of 21mer RNA (RNA21) and DNA (DNA21) are shown in Table S1. (B) Synthesis of **BiBA-AI** in DMSO and characterization with ^{1}H NMR spectroscopy. ^{1}H NMR (500 MHz, DMSO-d_6) δ 8.58 (s, 1H), 7.88 (t, *J* = 1.6 Hz, 2H), 7.74 (s, 3H), 7.10 (d, *J* = 0.9 Hz, 1H), 7.06 (d, *J* = 0.9 Hz, 6H), 2.09 (s, 16H). (C) Hydrolysis of **BiBA-AI** in 20% *v*/*v* DMSO in D_2O.

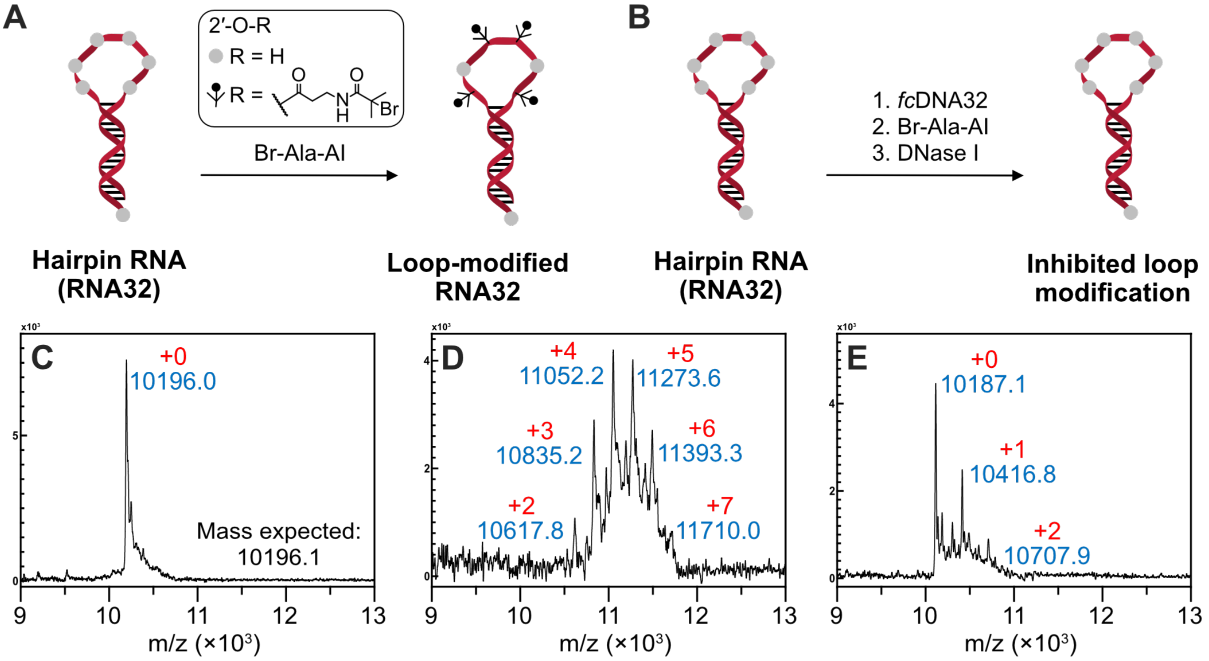

Figure S5. Hairpin RNA modification with **Br-Ala-AI**. (A and B) Scheme for RNA32 functionalization with **Br-Ala-AI** (A) with; and (B) without 2′-OH protection using fully-complementary DNA (*fc*DNA32), respectively. (C–E) MALDI-TOF spectrum of (C) untreated hairpin RNA; (D) **Br-Ala-AI**-treated hairpin RNA, and (E) **Br-Ala-AI**-treated hairpin RNA protected by *fc*DNA32 prior to treatment. 32mer hairpin RNA (RNA32) was used as a substrate. The single-stranded loop in the RNA32 is 4 nucleotides long flanked by 2 GU wobble pairs. The acylation efficiency for RNA32 was slightly higher (approximately 65%) than for RNA21 (approximately 30%). This difference in the efficiency may be attributed to the difference in the number of accessible 2'-OH groups present in the respective RNA sequences which affects the ratio of acylating reagent to 2'-OH groups: *ca.* 143:1 for RNA21; and *ca.* 391:1 for RNA32.

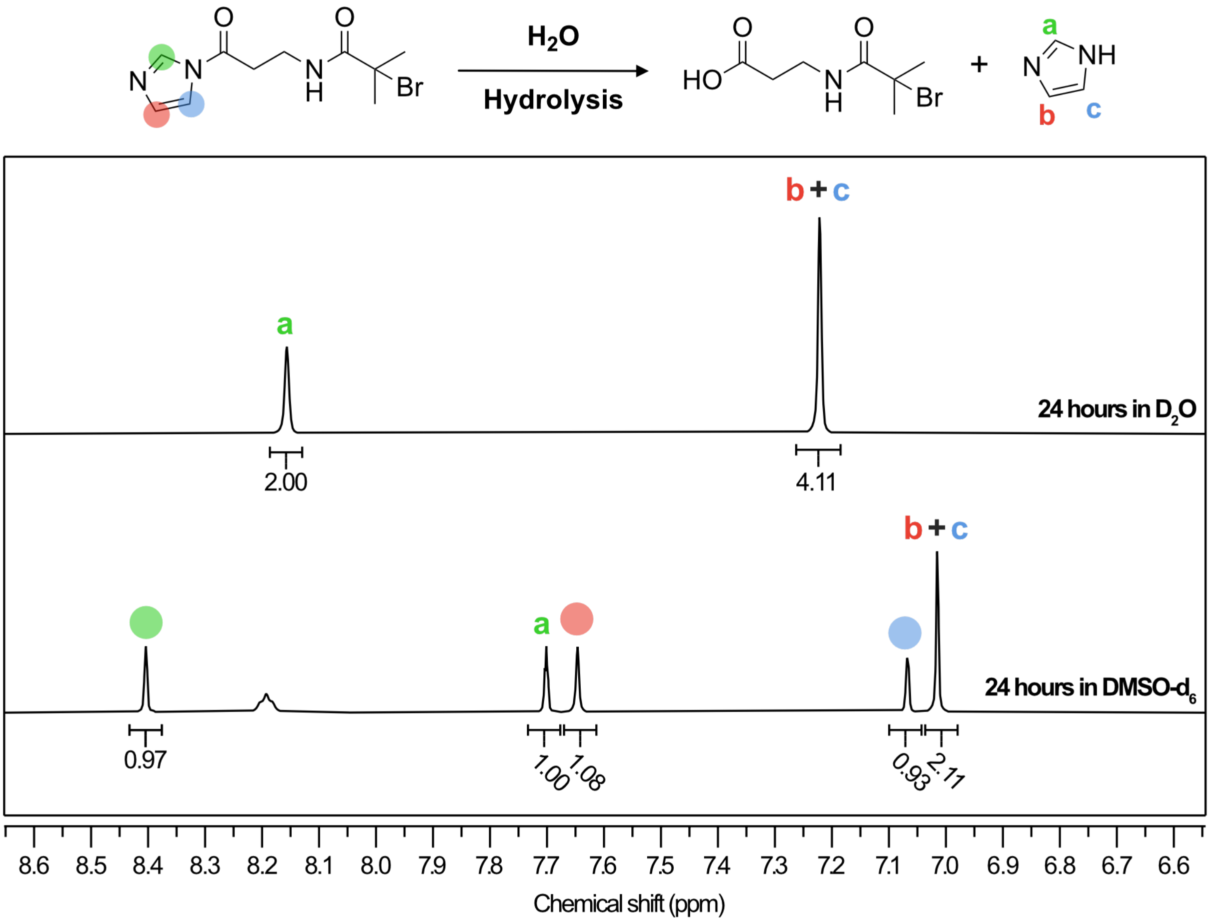

Figure S6. ^{1}H NMR spectra of **Br-Ala-AI** after 24 hours of incubation in (*Top*) D_2O and (*Bottom*) DMSO-d_6, respectively.

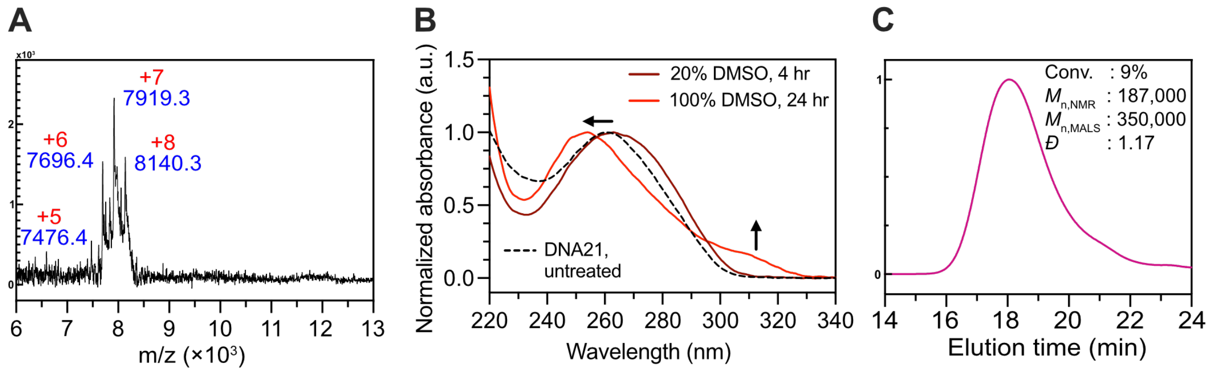

Figure S7. Water-free functionalization of DNA. (A) MALDI-TOF spectrum of 21mer DNA substrate (DNA21) after 24 h of water-free acylation in anhydrous DMSO. (B) UV-Vis spectra DNA21 after **Br-Ala-AI** treatment under different reaction conditions. (C) SEC-MALS trace of pOEOMA$_{500}$-grafted DNA21 prepared by water-free functionalization method. The polymerization product grafted from RNA21, synthesized in 100% DMSO, was too viscous for injection and SEC analysis.

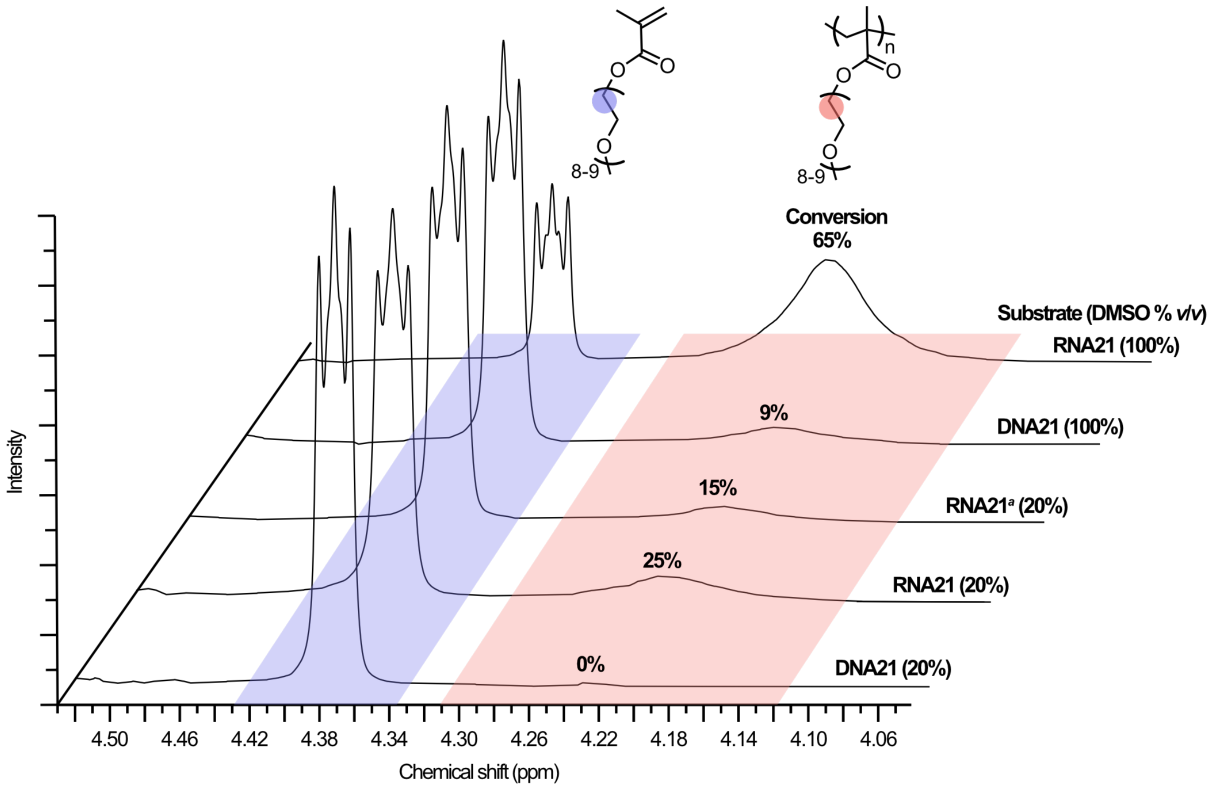

Figure S8. ^{1}H NMR spectra of polymerization products using initiators prepared under different conditions. Blue and red-colored areas correspond to protons in $OEOMA_{500}$ monomer (4.35–4.43 ppm) and polymerized $OEOMA_{500}$ (4.12–4.31 ppm), respectively. The polymerization results are shown in Table S2. [a] *pc*DNA21 was used as the helper DNA during the acylation process.

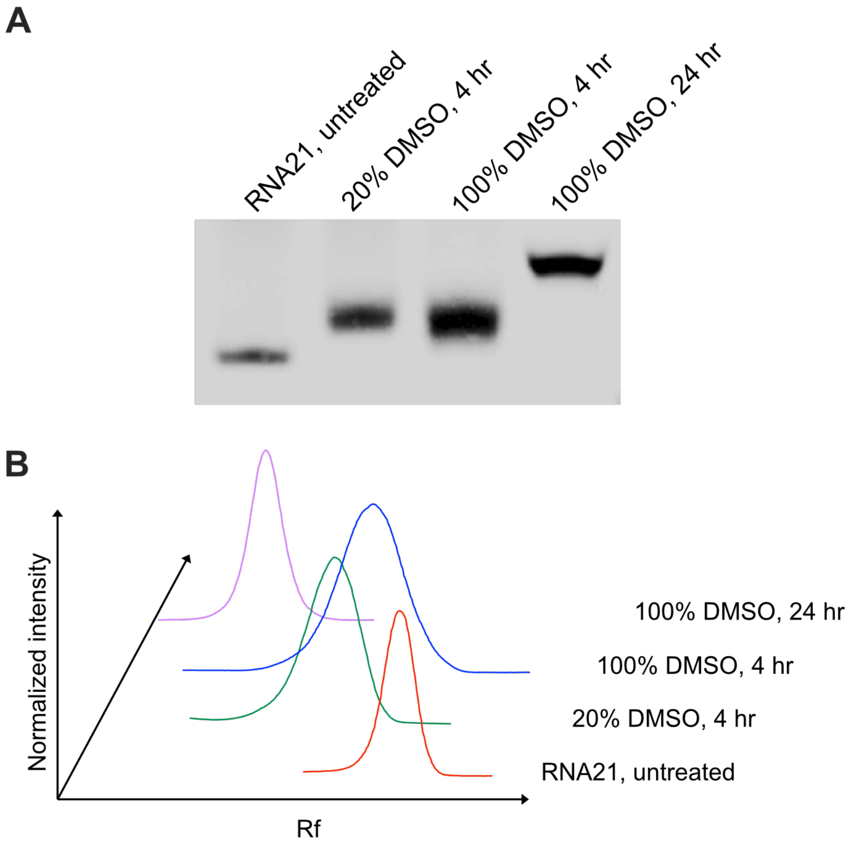

Figure S9. (A) PAGE of RNA21 after **Br-Ala-AI** treatment under different reaction conditions. PAGE condition: 12% native polyacrylamide gel was used at 120 V for 60 min due to short length of the oligonucleotides (21-mer). SYBR Gold was used for visualization. (B) Lane profiles of the bands in (A).

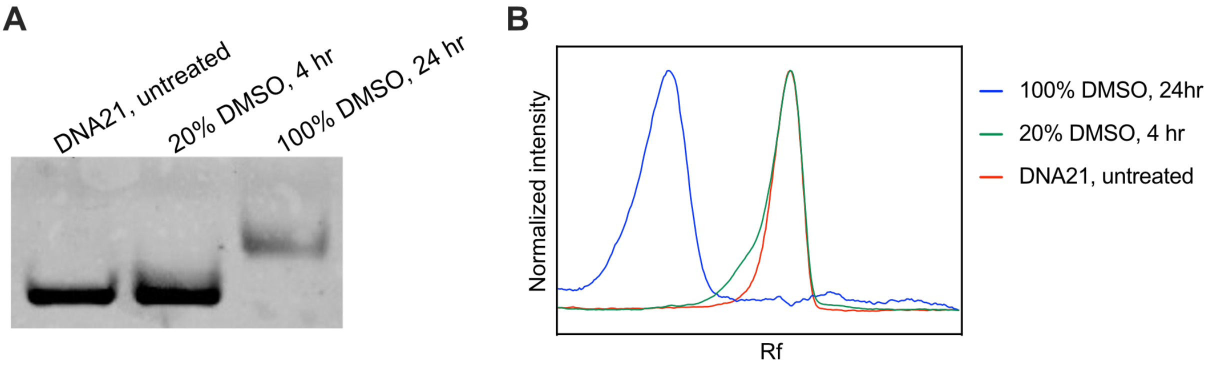

Figure S10. (A) PAGE of DNA21 after **Br-Ala-Al** treatment under the different reaction conditions. PAGE condition: 12% native polyacrylamide gel was used at 120 V for 60 min due to short length of the oligonucleotides (21-mer). SYBR Gold was used for visualization. (B) Lane profiles of the bands from (A).

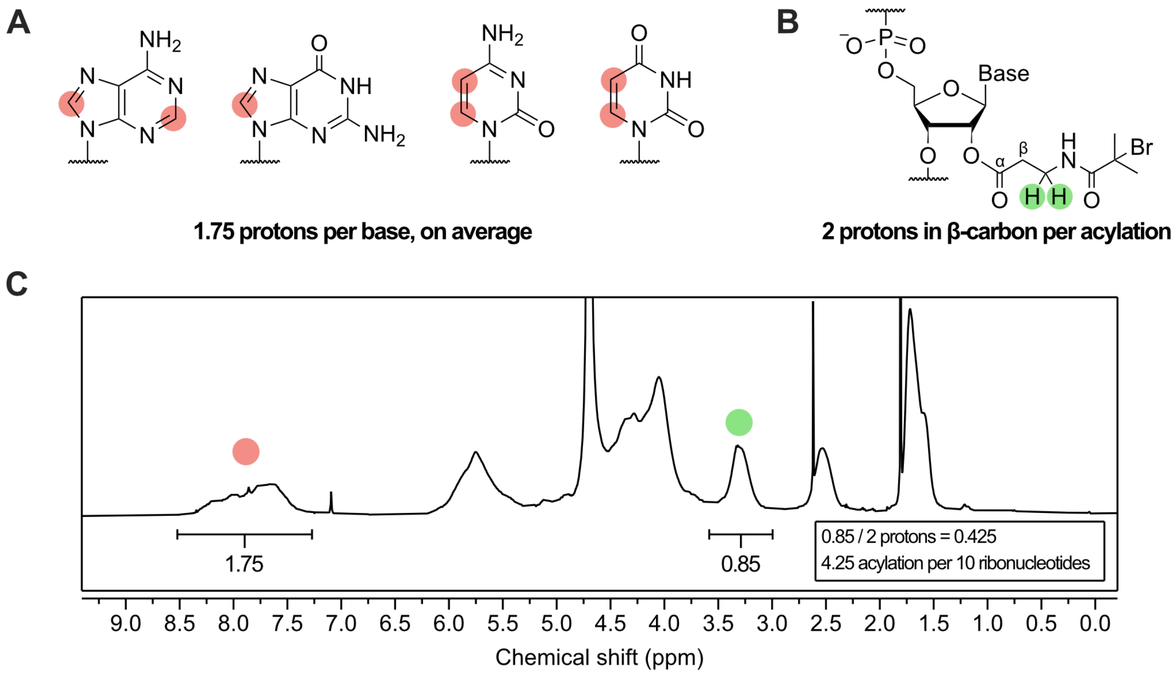

Figure S11. Estimation of the degree of acylation on *bm*RNA after the treatment of **Br-Ala-AI** overnight in 100% DMSO. (A) Molecular structure of the four RNA bases. The orange circles indicate the protons in the NMR spectrum within the range of 7.2–8.5 ppm. (B) A scheme of ATRP initiator-functionalized ribonucleotide. The green circles represent the two protons in the NMR spectrum within the range of 2.95–3.55 ppm. (C) ^{1}H NMR spectrum of *bm*RNA synthesized under the water-free condition. To estimate the ratio between ribonucleotides and ATRP initiator residues, the area under the peak corresponding to RNA bases was normalized to 1.75, representing the average number of protons in each RNA monomer unit. Next, the area of the NMR peak within the range of 2.95–3.55 ppm, corresponding to the two protons in the β-carbon of the initiating residue on the RNA, was obtained and divided by 2. This gives the amount of acylation per ribonucleotide (0.425 acylation per ribonucleotide)

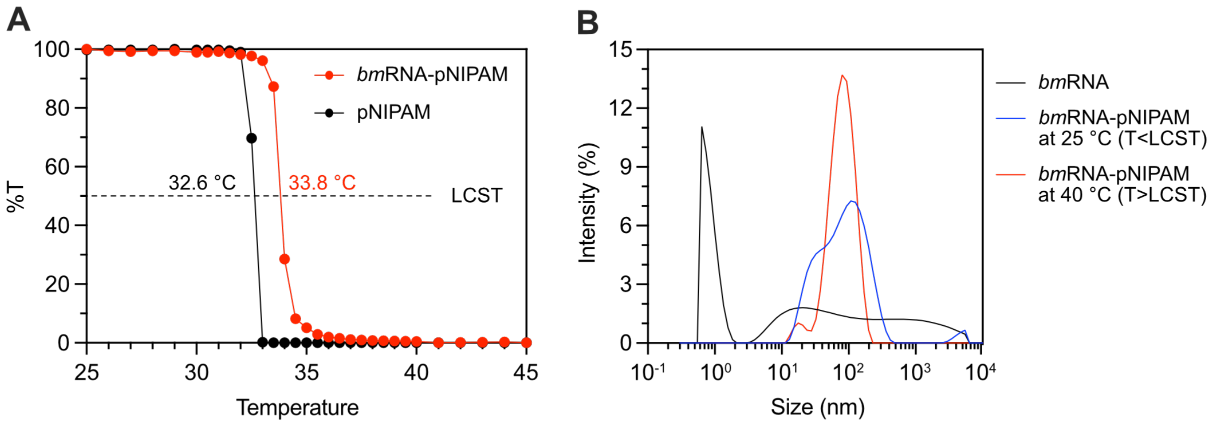

Figure S12. (A) LCST transition of *bm*RNA-pNIPAM conjugate in water. As the control group, pNIPAM initiated from a conventional ATRP initiator (HEBiB, 2-hydroxyethyl α -bromoisobutyrate) was used. Slightly increased LCST of bmRNA-pNIPAM may be attributed to the presence of the hydrophilic RNA motif, which hinders the precipitation of the pNIPAM block. (B) Characterization of *bm*RNA-pNIPAM using DLS. $M_{n,NMR}$ of NIPAM chain grafted from the bmRNA = 68,500. $M_{n,NMR}$ of NIPAM polymerized from HEBiB = 71,600. Reaction condition: [NIPAM]/[EYH_2]/[$CuBr_2$]/[Me_6TREN] = 1000/0.045/2.7/8.1, [*bm*RNA] = 0.5 mg/mL and [NIPAM] = 1000 mM under green light irradiation (520 nm, 3.7 mW cm^{-2}) for 30 min, in 80% *v*/*v* DMSO in PBS. For the synthesis of control pNIPAM, HEBiB was used as the initiator at the final concentration of 1 mM, instead of *bm*RNA initiator. ^{1}H NMR spectroscopy was used for the determination of NIPAM conversion using DMF as the internal standard (Figure S15).

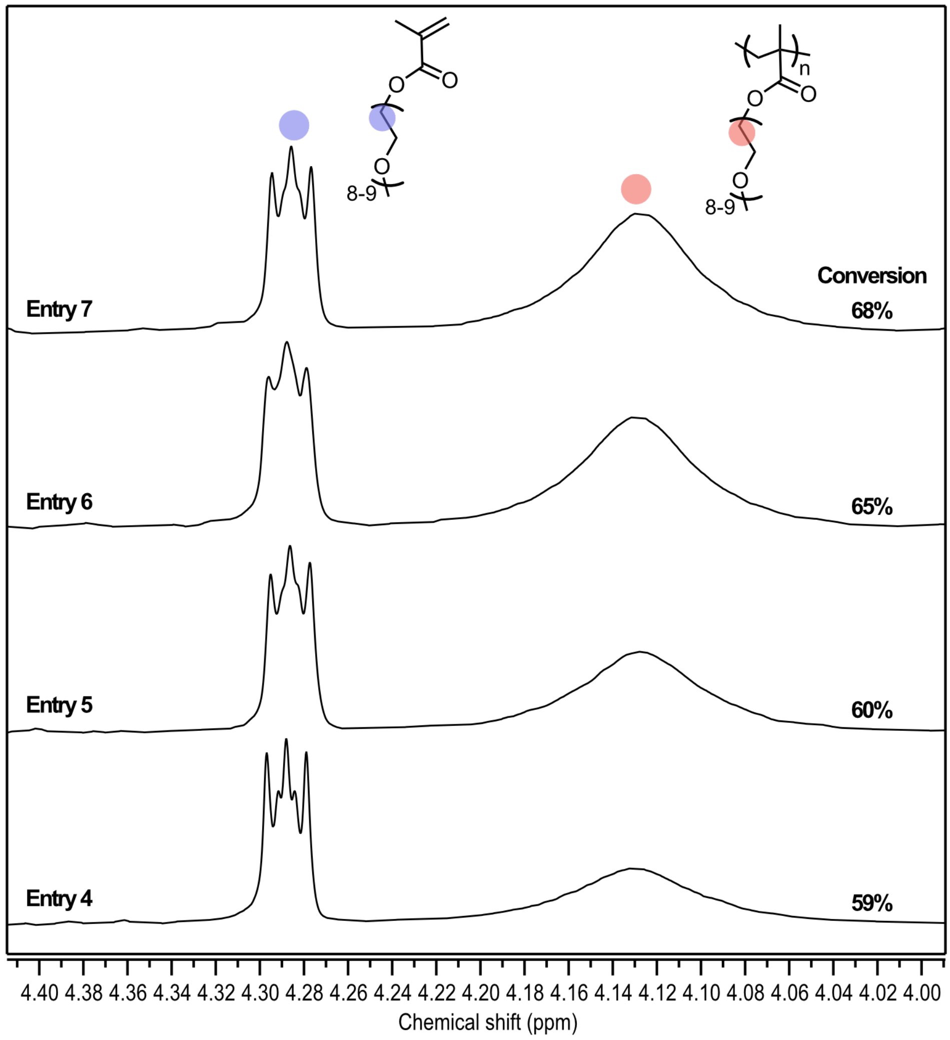

Figure S13. ^{1}H NMR spectra for the determination of the $OEOMA_{500}$ monomer conversion shown in the Table 1.

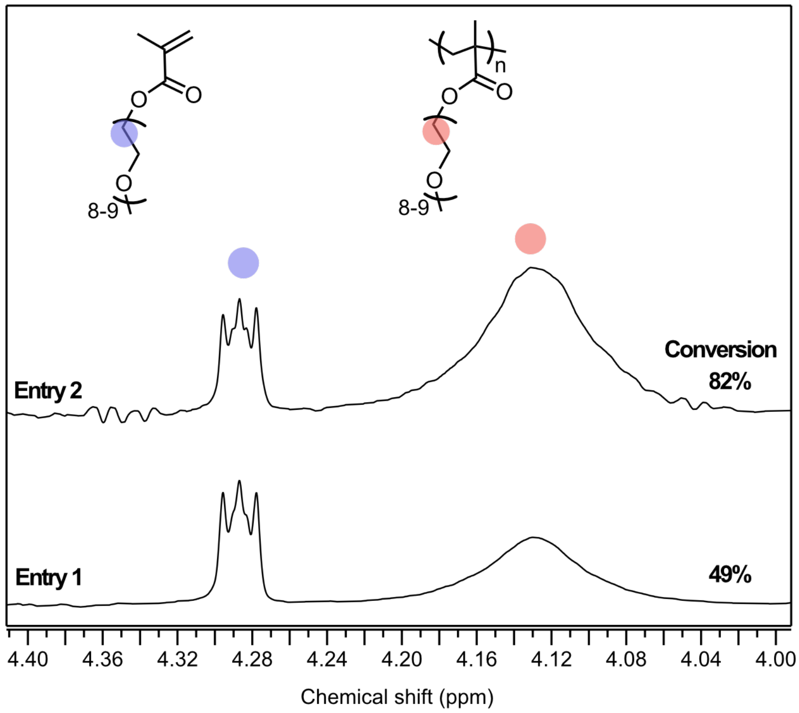

Figure S14. ^{1}H NMR spectra for the determination of $OEOMA_{500}$ monomer conversion shown in the Table S1.

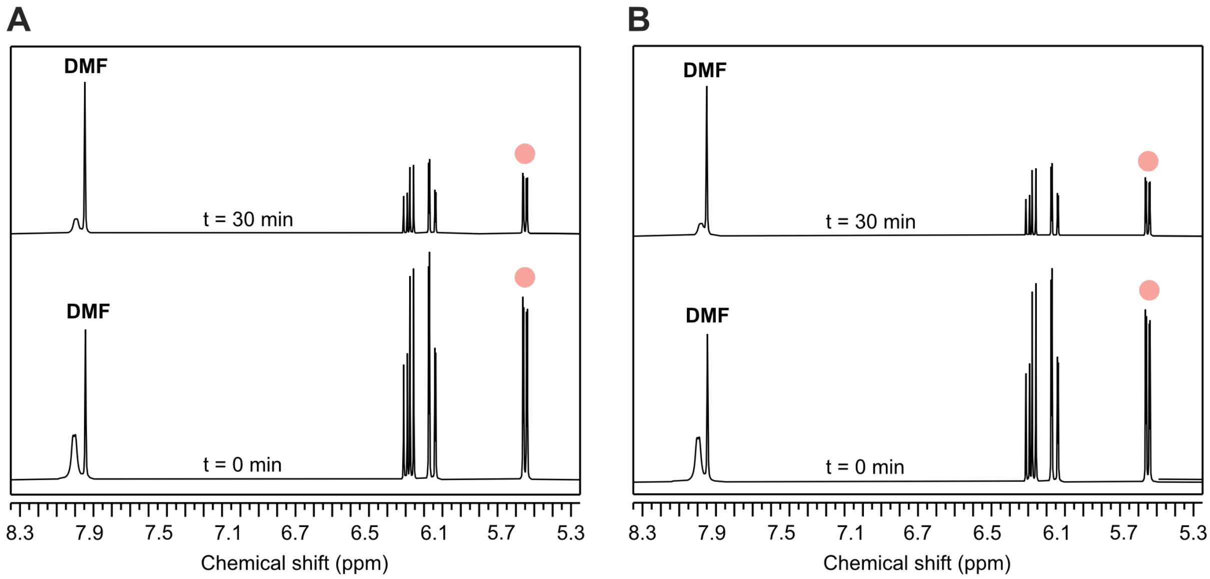

Figure S15. ^{1}H NMR spectra for the determination of the NIPAM monomer conversion. (A) *bm*RNA synthesized in water-free condition; and (B) HEBiB were used as the initiator, respectively.

Table S1. Sequences of oligonucleotides used in this study.

Name	Sequence (5' to 3')
RNA21	rArCrA rGrCrU rCrUrG rArCrU rGrCrU rCrGrA rCrGrU
DNA21	ACA GCT CTG ACT GCT CGA CGT
*fc*DNA21	ACG TCG AGC AGT CAG AGC TGT
*pc*DNA21	ACG TCG AGC TGT CAG AGC TGT
RNA32	rGrArA rGrArU rGrGrU rGrCrG rGrGrU rUrUrU rUrUrC rGrCrA rCrCrA rUrCrU rUrC
*fc*DNA32	GAA GAT GGT GCG GGT TTT TTC GCA CCA TCT TC

Table S2. Summary of grafting from oligonucleotide initiators.

Entry	Oligonucleotide	Acylation condition	Initiator per oligo[a]	DP[b]	Conv.[c]	$M_{n,NMR}$ (kDA)	$M_{n,MALS}$ (kDA)	$Đ$
1	RNA21	20% DMSO, 4 hr	6	924	25%	487	470	1.17
2	DNA21	20% DMSO, 4 hr	0	N/A	0%	N/A	N/A	N/A
3	RNA21[d]	20% DMSO, 4 hr	1.5	836	15%	307	425	1.13
4	RNA21	100% DMSO, 24 hr	23	N/A	65%	1,307	Too viscous	N/A
5	DNA21	100% DMSO, 24 hr	7	684	9%	186	350	1.15

[a]The number of initiators per oligonucleotide was calculated from the corresponding mass spectrum. [b]The degree of polymerization (DP) was calculated using the following equation ($M_{n,MALS}$ of the oligonucleotide-polymer conjugate – molar mass of oligonucleotide initiator) divided by 500, the molar mass of $OEOMA_{500}$. [c]The conversion was determined by ^1H NMR (Figure S8). [d]*pc*DNA21 was introduced as the helper DNA prior to acylation.

Table S3. Result of chain extension experiments from Figure 6F.

Entry	Initiator	Initiator concentration	Conv.[a]	$M_{n,MALS}$ (kDA)	$Đ$
1	Biomass RNA (*bm*RNA)	6 mg/mL	66%	146.7	1.32
2	*bm*RNA-pOEOMA block (From Entry 1)	3 mg/mL	49%	329.7	1.44

[a]Conversion was determined by ^{1}H NMR spectroscopy as shown in the Figure S14.

Reference

(1) Murata, H.; Cummings, C. S.; Koepsel, R. R.; Russell, A. J. Polymer-based protein engineering can rationally tune enzyme activity, pH-dependence, and stability. *Biomacromolecules* 2013, *14* (6), 1919-1926.
